# Public Perceptions Toward Community Management Policies for Individuals Convicted of Sexual Offenses: A Systematic Review and Meta-Analysis

**DOI:** 10.1177/15248380231174695

**Published:** 2023-05-18

**Authors:** Olga Sánchez de Ribera, Larissa S. Christensen, Nicolás Trajtenberg, Kirsty Hudson

**Affiliations:** 1 The University of Manchester, UK; 2 University of the Sunshine Coast, Sunshine Coast, QLD, Australia; 3 Cardiff University, UK

**Keywords:** public perceptions, sexual offenses, community policies, systematic review, meta-analysis, child sex offenders

## Abstract

Community management policies for individuals convicted of sexual offenses (ICSO) are controversial, mainly because the effectiveness of these policies in reducing recidivism is limited and appear to have some collateral effects. Despite this, the current meta-analysis found the public highly support these policies. Studies examining public perceptions regarding community management policies for ICSO to understand levels of support, misconceptions about the policies, and factors affecting the views of the public were reviewed. After searching 7 electronic databases, 43 studies, both quantitative and qualitative, were included in the systematic review and 31 in the meta-analysis. Studies could be longitudinal or cross-sectional, needed to include public attitudes, opinions, and perceptions about ICSO community management policies and could include standardized or non-standardized measures, indirect assessments of attitudes, along with interviews and focus groups. Results suggest that overall, policies were supported by 76% of the public, 61% believed in their effectiveness, and 63% felt safer because of the policies. However, only 36% accessed the registry, 38% took preventive actions, and 40% were aware/concerned about the collateral consequences. All analyses yielded high levels of heterogeneity. Misconceptions about policies and ICSO were moderate. Finally, 36 studies explored factors that affected the public’s attitudes and perceptions of policies with a variety of significant associations and predictors. The findings provide comprehensive evidence that while these policies are supported by the public, the public have less belief in the effectiveness of them in protecting children and reducing recidivism. Implications for public policy and future research are discussed.

Sex crimes produce great fear among citizens, especially when victims are children (Kernsmith et al., 2009; Levenson et al., 2007). These feelings are often perpetuated through the reporting of high-profile cases in the media (Wright, 2003). In order to minimize these public sentiments concerning individuals convicted of sexual offenses (ICSO) released in the community, policymakers intend to protect the public by applying additional punitive, rather than rehabilitative, measures (Baker et al., 2015). Although the extent of these policies differs from country to country, the most common community management policies include offender registration, community notification through public online registries, residency restrictions, electronic monitoring, and chemical castration. Offender registration requires that ICSO register their address with the local enforcement department. This sanction can last a minimum of 10 years (see Comartin et al., 2009). Community notification typically involves public disclosure of the identity and address (and other information) of the ICSO through mass media (i.e., Internet, TV, and newspapers), flyers/posters, and community meetings. Electronic monitoring involves tracking the location (but not the individual’s activities) of the ICSO using Global Positioning System (GPS) monitoring. Finally, chemical castration requires pharmacological treatment to reduce the sexual arousal to decrease sexual reoffending (see Comartin et al., 2009). The main goal of these polices is to protect the public from sexual offenses by tracking ICSO (i.e., work and residence) to ultimately decrease sexual recidivism (Kernsmith et al., 2016).

Community management policies for ICSO have raised some contentious points. First, these policies have been found to have little or no effect on deterring sexual offenses (Duwe et al., 2008; Zevitz & Farkas, 2000) with difficulties preventing reoffending, despite this being the main aim of these policies (Kernsmith et al., 2016). Second, these laws increase the level of fear and anger in some residents (Bedarf, 1995; Phillips, 1998; Zevitz & Farkas, 2000), bringing with it some negative consequences for ICSO such as harassment, threats, property damage (Tewksbury & Lees, 2006), housing instability and reduced or lost employment opportunities (Mercado et al., 2008; Tewksbury & Mustaine, 2009), and living in areas with great social disorganization (Mustaine et al., 2006). Thus, these collateral consequences have been argued to not only violate ICSO constitutional rights such as offenders’ rights to privacy and dignity, and risk offenders being punished twice for the same crime, and subject to cruel and unusual punishment (Bedarf, 1995; Redlich, 2001), but also prevent reformed ICSO from reintegration (Bedarf, 1995). However, the purpose of the current study is not to discuss the evidence on the effectiveness of these policies. Instead, the focus is on understanding public perceptions of these policies, misconceptions, and the factors associated with perceptions. Such research might help to inform implementation—and promotion—of more suitable alternative policies. In turn, potentially reducing collateral consequences of sanctions on ICSO and positively impacting rehabilitation and reintegration efforts.

## Public Support for Community Management Laws

Public policies are strongly influenced by public opinions rather than research evidence (Burstein, 2003; Center for Sex Offender Management [CSOM], 2010). Studies have consistently reported the misconceptions and lack of knowledge the public have about ICSO (e.g., level of risk, recidivism, and treatment) and community management laws (for review, see Zatkin et al., 2021). As previously noted, despite current evidence against the effectiveness of some of these policies, these sanctions are very popular among public opinion. For example, Levenson et al. (2007) found that regardless of whether there is scientific support for an instrumental effect of community notification, the public supports such policies. This finding was corroborated more recently by Koon-Magnin’s (2015) study using an experimental design. Phillips (1998) found that 80% of Washington State residents believed that community notification was very important. Other studies found that community notifications in different countries were perceived as fair by the public (Brannon et al., 2007; Levenson et al., 2007; Schiavone & Jeglic, 2009; Zgoba & Cowan, 2020). Importantly, one study showed that support for ineffective policies persists even when explicit information on the lack of effectiveness of these laws in reducing crime was provided (Campbell & Newheiser, 2019). Such findings are concerning when public attitudes have a direct influence on policymakers, who follow public opinion sentiments when making decisions (CSOM, 2010; Meloy et al., 2013).

## Attitudes Toward Community Management Laws

The public hold strong attitudes for community management policies, with the reasons for, and motivations behind, such attitudes varying greatly. However, the first reason might be the type of crime. Evidence has yielded negative attitudes toward ICSO, and all types of sexual offenses have been found to elicit some fear from respondents (Higgins & Ireland, 2009; Kernsmith et al., 2009; Levenson et al., 2007). Second, attitudes may be influenced by the media, which has been found to cover predominantly sensationalized crimes involving child abductions and murders such as Megan Kanka in the United States and Sarah Payne in the United Kingdom (Kernsmith et al., 2009). Often, the ICSO are portrayed as “monsters” and “deviants” (Quinn et al., 2004). These laws hold symbolic value among the public, promoting a sense of public safety (Koon-Magnin, 2015). Third, evidence suggests that the public’s beliefs about ICSO may be based upon myths and misconceptions (Harper et al., 2017), which have been associated with a high level of punitiveness among community members (King, 2019). Finally, the consequentialism rather than the instrumentalism approach may play a role, that is, people want to punish ICSO because they are deserving and this “additional” punishment after prison is fair (Schiavone & Jeglic, 2009; Zgoba & Cowan, 2020).

## The Current Review

Given that public attitudes can have major implications on the decisions made by policymakers (Levenson et al., 2007), understanding public attitudes associated with ICSO policies is essential for rehabilitation and reintegration. Furthermore, understanding such attitudes is also ideal in order to design—and promote—alternative strategies, which may be perceived as less popular among the public but may utilize more evidence-based measures. While research has reviewed professionals’ perceptions of such policies (see Christensen et al., 2021), to the best of the authors’ knowledge only one published review examined attitudes of different populations, which included the general population from North America (Connor & Tewksbury, 2017). With regard to perceptions of the public, they found that Americans largely endorsed notification and registration laws and considered them as fair strategies to manage ICSO even though the public recognized the drawbacks ICSO faced from these laws. However, this was not a systematic review. Moreover, it solely included studies on ICSO notification and registration laws in the United States, excluding other countries and other laws such as residency restrictions.

The current study offers a systematic review and meta-analysis, which focuses on the publics’ perceptions toward ICSO community management policies described above, and it has three main aims: (**1**) to synthesize the evidence on the publics’ perceptions toward ICSO community notification laws and, where possible, to calculate the overall proportion of the population who supported these laws; (**2**) to examine whether the public hold misconceptions about these laws; and (**3**) to examine what factors impact on these attitudes and, where possible, to calculate the proportion of other factors associated with these policies. The current study offers a valuable resource for researchers, policy professionals, along with government and non-government organizations who research or manage community management policies for ICSO.

## Methods

This review adhered to the Preferred Reporting Items for Systematic Review and Meta-Analysis (PRISMA, Moher et al., 2009).

### Eligibility Criteria

To be included in this review, the studies had to meet the following criteria. First, studies included the general public of any age group, gender, and nationality. Studies focused on practitioners of the criminal justice system (for review, see Christensen et al., 2021), ICSO, family of ICSO, and media were excluded. Second, studies needed to include attitudes, opinions, and perceptions about ICSO community management policies. The studies had to include standardized measures (i.e., Attitudes Toward Sexual Offenders, Community Attitudes Toward Sex Offenders), nonstandardized measures (e.g., surveys and vignettes), and indirect assessments of attitudes toward ICSO and community policies (see Harper et al., 2017), along with interviews and focus groups. Studies focused merely on attitudes toward ICSO and not referring to any community management policy were excluded. Third, the study design could be either longitudinal or cross-sectional. If cross-sectional studies compared two different groups (e.g., community members and students), information from the groups was analyzed separately, but practitioners were excluded. Fourth, the studies could be from any country, as long as they were published in English and or Spanish languages. Lastly, books, book chapters, newspaper articles, and conference abstracts were excluded, but peer-reviewed articles, government reports, official reports from non-government agencies or other organizations, and dissertations were all included.

### Search Strategy

The search for eligible studies was conducted using three types of keywords using Boolean operators relevant to: (**1**) ICSO (i.e., sex* offen* OR child sex* offen* OR child sex* abuser OR sex* crim* OR child molest* OR paedo* OR pedo* OR rap* OR sex* predator*), (**2**) attitudes (i.e., public OR opinion OR perception* OR attitude* OR judgement* OR judgment* OR myth* OR misconception* OR stereotyp* OR bias OR belief OR decision*), and (**3**) community management policies for ICSO (i.e., regist* OR notification OR law OR legislation OR Megan’s Law OR policy OR reintegration OR management OR punitive* OR risk) in seven electronic databases (Medline, PsycNet, Scopus, Web of Science, OpenGrey, Informit, and SciELO) published between January 1994 and October 2019. This search was conducted as part of a larger study. In addition, all of the reference lists of the studies included for analysis were reviewed.^1^

### Study Selection

**Figure 1.**
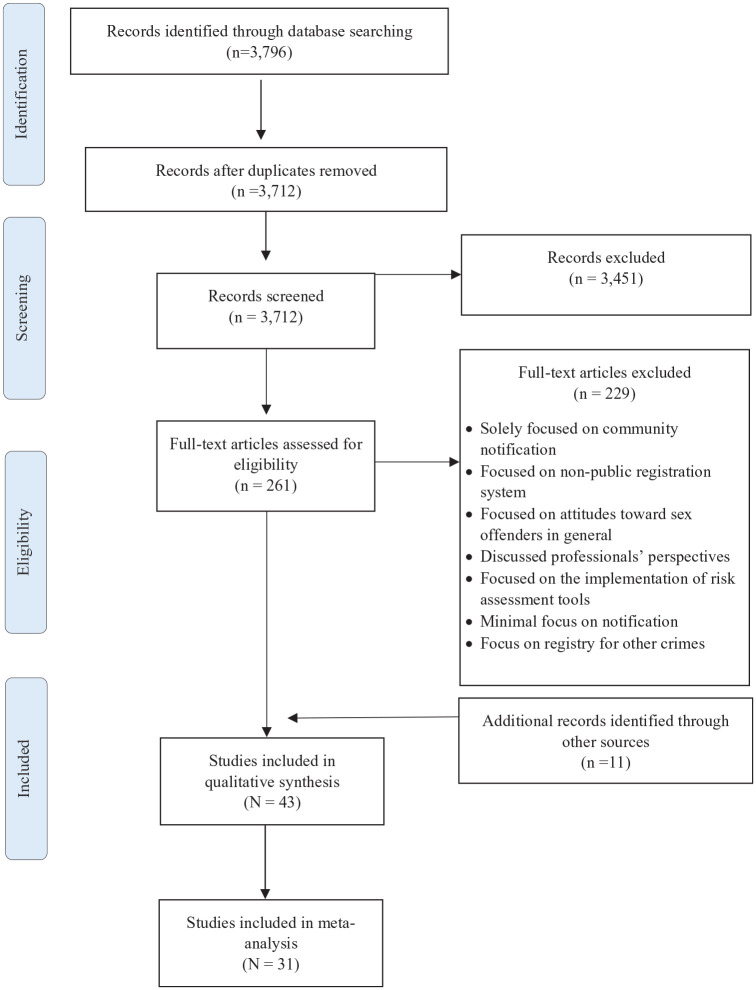
Preferred Reporting Items for Systematic Review and Meta-Analysis (PRISMA) 2009 flow diagram.

The search strategy yielded a total of 3,796 document references. After deleting duplicates and an initial screening of titles and abstracts, 261 studies were potential candidates to be included. A total of 223 studies were excluded, after assessing the full text. Finally, a total of 43 studies met the inclusion criteria of the review, and 31 studies were included in the meta-analysis. The search strategy and the selection process were conducted by a researcher and supervised by the second author. Figure 1 presents a flow diagram of the selection process.

### Data Management, Extraction, and Analysis

Standardized and piloted data extraction forms were used to record all relevant data for each included study. For all studies, the authors extracted information regarding the study characteristics, sample characteristics, methodology, and outcomes. One reviewer (first author) extracted data on 33 variables. Additionally, the coding process involved identifying common categories of questions relevant to the dependent variable (public perceptions of support of community management laws and awareness of negative consequences of these laws for ICSO). When questions were asked in more than five studies, the overall proportion of these questions was calculated. The responses were dichotomized ([Strongly] Disagree/[Strongly] Agree) and the effect sizes were reported as proportions *n*/*N* (%). The transformation for proportions used was the arcsine-square-root, which stabilizes the variance and makes it dependent on just the sample size, not the value of the proportion itself (Barendregt et al., 2013).

In all meta-analyses, a random-effects analysis was employed based on the assumption that studies use varied research designs and take samples of observations from different populations, so this model assigns similar weights to all studies regardless of sample size (Borenstein et al., 2011). The level of heterogeneity was measured using the *Q-*static and *I*^2^ (*I*^2^ > 75%), which indicated high significant heterogeneity between studies (Higgins & Thompson, 2002). Categorical moderators (i.e., sample and policies, and quality) were assessed with the *Q-*between statistic (*Q*_b_). Continuous moderator (i.e., year) was assessed using the meta-regression (random model). All statistical analyses were performed using Open Meta-Analyst software (Wallace et al., 2012).

### Quality Appraisal of Studies

The Quality of Survey Studies in Psychology (Q-SSP) checklist (Protogerou & Hagger, 2020) was used for the quality appraisal of studies. The Q-SSP uses several responses (“Yes,” “No,” “Not stated clearly,” and “Not applicable”) and contains 20 items grouped into four domains: Introduction (rationale/variables: 4 items), Participants (sampling/recruitment: 3 items), Data collection/analyses/measures/results/discussion: 10 items), and Ethics (3 items). If the overall quality score is ≥75%, the study may be considered to be acceptable quality. Two reviewers independently extracted data (third author and fourth author). To ensure intercoder reliability, the two reviewers practiced coding and established procedures for resolving ambiguities. The rater agreement was 83% (κ = .66).^2^ Disagreements were resolved by discussion, and always reached a consensus.

## Results

Results from the 43 studies and 31 studies that met the inclusion criteria for the systematic review and meta-analysis, respectively, are discussed below. Study characteristics are first discussed, followed by a critical appraisal of the included studies. The results are then presented in three parts based on the findings of the meta-analysis: (1) public perceptions toward community management policies for ICSO, (2) knowledge and misconceptions about community management policies for ICSO, and (3) factors contributing to attitudes and perceptions of community management policies for ICSO.

### Study Characteristics

The demographic information and sample characteristics of the studies included in this review are presented in Supplemental Appendix A. The majority of studies were conducted in the United States (*k* = 39, 89%) and collected samples of adults from the general public (*k* = 39, 89%), with a higher proportion of females with different age ranges comprising a total of 29,515 respondents. More than half of the studies (*k* = 24, 54%) employed a randomization sampling technique (e.g., random digit dialing and random addresses), and less than half of the studies used online surveys (*k* = 13, 30%). The response rate ranged from 11.4% to 69%, but 23 studies neither reported the response rate nor the authors were able to calculate it.

### Critical Appraisal of Included Studies

The overall quality scores for each study using the Q-SSP checklist is presented in Supplemental Appendix B. The studies did not meet the score for acceptable quality (i.e., score >75%). The most common biases across the studies were on the ethics and data domains.

### Public Perceptions Toward Community Management Policies for ICSO

Studies explored a wide range of variables on public perceptions toward different community policies for ICSO. The most common variables explored among the studies were: support for community management policies of any type of ICSO including females who committed sex offenses (*k* = 24, 56%), effectiveness/usefulness of laws in reducing sexual offending and protecting children (*k* = 18, 41%), access to the registry (*k* = 8, 19%), feeling of safety for themselves, family and the community (*k* = 12, 28%), awareness and concern of vigilantism/negative consequences for ICSO (*k* = 11, 25%), taking preventive actions (*k* = 8, 19%), familiarity with community management laws (*k* = 9, 21%), fear of having a ICSO in the community (*k* = 8, 19%), rehabilitation views (*k* = 8, 18%), research influence on their policy views/support (*k* = 5, 12%), useful information in the registry (*k* = 5, 12%), and policy fairness (*k* = 4, 9%) (see Supplemental Appendix C).

Other topics examined by less than five studies involved fairness of policies, most effective type of notification method, deserving the registration/notification, awareness of released ICSO in the community, information in the registry, number of times/reasons for accessing the registry, notification should be available to the public, the ways public found out about the disclosure scheme, reactions to community notification, police do a good job of notifying citizens about released ICSO, police deal appropriately with citizens’ reactions to released ICSO (included in only two studies), and neighborhood characteristics.

#### Meta-analysis

**Figure 2.**
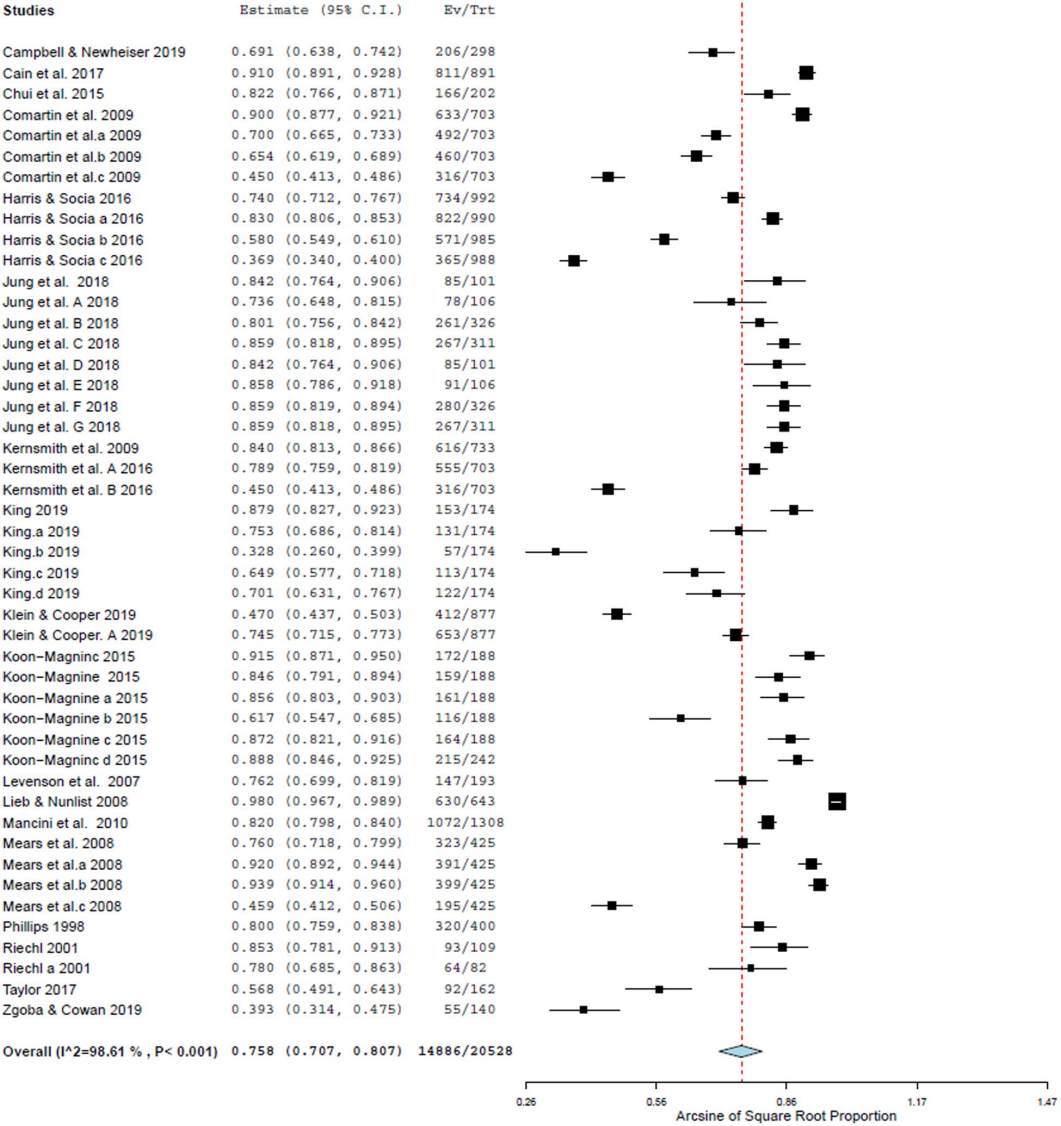
Forest plot of the overall prevalence of supporting these policies.

Thirty-one studies (out of 43) were included in the meta-analysis (see Supplemental Appendix C). The overall pooled proportion estimate of respondents’ support for different policies was 76% (95% confidence interval [CI] = 71–81), with very high levels of between-study heterogeneity (*Q* = 3296.03, *p* < .001, *I*^2^ = 99%) (see Figure 2).

Using subgroup analysis, possible explanations for between-study variation were investigated when it was possible and meaningful to do so (see Table 1). Internet and social network ban were the least supported policies (47% [95% CI = 27–68]), whereas severe policies such as life/long prison and castration yielded considerable support (66% [48–82]). Notification (85% [76–93]), registration (81% [76–86]), and residence restrictions (80% [74–85]) yielded similarly high levels of support, as well as controlling movement using GPS (73% [59–84]). Additionally, similar levels of support were found for community members (76% [70–81]) and students (78% [68–86]) with higher levels for the latter. Regarding familiarity of these community management policies and awareness of the registry, more than half of the residents were familiar (67% [54–78]) with high heterogeneity (*Q* = 753.75, *p* < .01, *I*^2^ = 98.5%) due to sample and type of policy. Community residents (69% [ 56–81]) were more familiar than students (53% [32–74]). When exploring differences among policies, a high proportion of respondents were aware of the registry (72% [54–87]) than familiar with community management laws (63% [47–77]).

**Table 1.** Pooled Proportions Estimates for Most Common Categories by Subgroups (Sample, Policies).

Sample and Policies	Policy Support % [95% CI], *Q*, *I*^2^	Effectiveness/Utility % [95% CI], *Q*, *I*^2^	Familiarity With Policies/Registry Awareness % [95% CI], *Q*, *I*^2^	Registry Access % [95% CI], *Q*, *I*^2^	Feeling Safer (Themselves, Family, Community) % [95% CI], *Q*, *I*^2^	Taken Preventive Actions % [95% CI], *Q*, *I*^2^	Awareness/Concern of Collateral Consequences of Policies % [95% CI], *Q*, *I*^2^
Sample							
Community members	76 [70, 81], 3255.64*, 99	65 [59, 70], 1083.87*, 97	69 [56, 81], 685.84*, 99	36 [29, 43], 154.66*, 97	68 [54, 80], 560.03*, 98	67 [50, 83], 1257.72*, 99	36 [25, 48], 299.75**, 98
Students	78 [68–86], 43.11*, 88	46 [32–61] 197.55*, 96	53 [32–74] 10.14*, 90	—	55 [33–76] 183.23*, 98	—	47 [31–63], 35.57**, 91.5
Policies							
Notification	85 [76–93], 321.72**, 98	75 [63–85] 100.74**, 96	—	—	74 [56–89] 215.35*, 97	37 [20–54] 1190.01*, 99	7 [4–11], NA
Registration	81 [76–86] 142.62**, 92	45 [28–63] 505.01**, 98	72 [54–87] 234.31*, 98	—	58 [43–72] 329.75*, 98	60 [55–64] NA	43 [33–54], 240.41**, 97
Notification and registration	56 [30–80] 206.20**, 99	68 [62–74] 69.93**, 91	—	—	60 [56–64] NA	—	44 [34–54], NA
Residence restriction	80 [74–85] 275.58*, 96	60 [53–66] 33.94**, 88	—	—	—	—	40 [31–50], NA
Notification, registration and residence restrictions	69 [64–74] NA	—	—	—	65 [55–74] NA	—	—
Movement control/GPS	73 [59–84] 35.93**, 94	76 [64–87] 36.82**, 94	—	—	—	—	—
Internet/social network ban	47 [27–68] 88.79**, 99	—	—	—	—	—	—
Life/long prison and castration	66 [48–82] 569.01**, 99	—	—	—	—	—	—
Prison	—	64[46–81] 65.96**, 97	—	—	—	—	—
Castration	—	54 [47–61] 1.99, 50	—	—	—	—	—
Supervision by parole/probation officers	—	74 [71–77] NA	—	—	—	—	—
Drastic: vigilantism, registration	—	39 [32–46] 0.005, 0	—	—	—	—	—
Overall	76 [71–81], 3299.90**, 99	61 [55–66], 1426.69*, 97	67 [54–78] 753.75*, 98	36 [29–43] 154.66*, 97	63 [52–74] 848.23*, 98	38 [23–55] 1257.72*, 99	40 [30–50], 353.65**, 97

*Note.* Fear was not included because only four studies reported data for meta-analysis. 95% CI = 95% confidence interval; NA = not applicable.

* *p* *<* .01. **p < .001.

Despite respondents’ showing high levels of support toward these policies, the belief on effectiveness or usefulness of these policies in reducing recidivism and protecting children was lower. The overall pooled proportion estimate was 61% [95% CI = 55–66], with very high levels of between-study heterogeneity (*Q* = 1426.69, *p* < .01, *I*^2^ = 97%; see Table 1). Policies reported as more effective were movement controlled by GPS (76% [64–87]), notification (75% [63–85]), and notification and registration (68% [62–74]). Registration alone was not seen as effective by respondents (45% [28–63]). All subgroups showed significantly high levels of heterogeneity except for castration, which 54% [47–61] of respondents believed it was effective. However, this result is based on only two studies, and therefore must be interpreted with caution. Another source of variation across the studies was the sample type, that is, community members believed these policies were more effective (65% [59–70], *I*^2^ = 97%) than students (46% [32–61], *I*^2^ = 96%).

The overall estimation for accessing the registry was low for community members (36% [95% CI = 29–43]) with significantly high levels of heterogeneity (*Q* = 154.66, *p* < .01, *I*^2^ = 97%). Overall, 63% [52–74] felt themselves, the family, and the community were safer with the notification and registration with high heterogeneity between the studies (*Q* = 848.231, *p* < .01, *I*^2^ = 98%). Both sample and policies were significant factors of heterogeneity. Community members (68% [54–80]) felt safer than students (55% [33–76]), and notification 74% [56–89] yielded a higher feeling of safety than registration 58% [43–72].

Interestingly, a low percentage of residents took preventive actions after accessing the registry or being notified that a ICSO was living in their neighborhood (38% [95% CI = 23–55], *Q* = 1257.72, *p* < .01, *I*^2^ = 99%). Additionally, the majority of respondents were not fully aware or underestimated the collateral consequences of different community management policies for ICSO (40% [30–50], *Q* = 353.65, *p* < .001, *I*^2^ = 97%). Significant differences were found for sample and policy types. Students (47% [31–63]) were slightly more aware of the negative consequences of registration than the community members (36% [25–48]). Less than half of respondents were aware or knew about the collateral consequences of the registry (43% [33–54]). Finally, the study’s publication year was not associated with the heterogeneity between the studies for any comparison.

### Knowledge and Misconceptions About Community Management Policies for ICSO

Twenty studies examined the level of knowledge that respondents had about issues related to recidivism rates and type, sexual offense rates, the offender–victim relationship, offenders’ civil rights and risk, treatment efficacy, views on policies even when there was no evidence, and information source (see Supplemental Appendix D). Policy effectiveness views and fairness perceptions were excluded because both were examined in the previous section (see Supplemental Appendix C). Since there was no available validated measure of knowledge about ICSO (Manchak & Fisher, 2019), studies generally used the statements of the CSOM or alternatively authors created their own measure, or a measure developed by other authors. Although studies found moderate levels of misinformation in general, it was difficult to estimate the level of knowledge since studies were very heterogeneous in reporting respondents’ perceptions and knowledge. Additionally, the mean total score for the Knowledge Questionnaire instrument varied across the studies such as Kernsmith et al.’s (2016; *M* = 2.41 [*SD* = 0.29]) and King’s (2019; *M* = 44.56 [*SD* = 8.29]) studies.

Overall, a high proportion of respondents were concerned that sex crime rates were rising, believed that ICSO have high recidivism rates (such as 50% or more), and viewed ICSO as more likely to reoffend than other types of criminals. Respondents seemed to have positive views on the treatment for ICSO, but they had a lack of knowledge on the level of risk as respondents typically suggested they would place all ICSO on the registry. One noteworthy finding was that generally well under half of respondents (30%) believed that many sex offenses are committed by strangers. Information about ICSO and policies is mainly obtained by television, and most respondents believed that media exaggerated the level of risk of ICSO and the risk of becoming a sexual abuse victim. Overall, half or more of the respondents would support the laws even if research showed otherwise, but 38% recognized that policymakers do what the public want even though 58% stated that policymakers should rely on the effectiveness of the laws. Other public misconceptions held by most respondents were: more than 80% agreed that one of the best ways to protect children from sexual abuse was teaching children about stranger danger (King, 2019), registration and notification were constitutional and did not violate ICSO privacy rights (Schiavone & Jeglic, 2009), and most registered ICSO were pedophiles or sexual predators (Socia & Harris, 2016).

### Factors Contributing to Attitudes and Perceptions of Community Management Policies for ICSO

Findings in this review suggest that factors affecting attitudes and perceptions of community management policies for ICSO are complex, diverse, and influenced inconsistently by a range of factors. Twenty-seven studies (out of 43, 63%) explored the role of factors, which may influence behaviors, misconceptions, and attitudes toward the policies for ICSO in the community (see Supplemental Appendix E). Eleven studies (41%) explored the correlation between variables, whereas other studies examined the role of different variables such as predictors (*k* = 21, 78%), moderators (*k* = 2, 7%), and mediators (*k* = 2, 7%).

Factors most commonly examined in the studies were demographic such as sex (*k* = 19, 70%), age (*k* = 15, 55%), race (*k* = 14, 52%), education (*k* = 15, 55%), income (*k* = 10, 37%), living area (*k* = 5, 18%), and marital status (*k* = 9, 33%). Other variables were parental status (*k* = 15, 55%), political ideology (*k* = 7, 26%), victimization (*k* = 8, 30%), fear (*k* = 2, 7%), religion (*k* = 3, 11%), neighborhood (*k* = 3, 11%), and views and knowledge on ICSO and the policies (*k* = 11, 41%). The significant associations of these variables across the studies are described below.

#### Sex

Twelve out of nineteen studies (63%) found a significant association with sex. Sex was significantly associated with knowing about the registry, the registry use, and taking preventive actions (Anderson & Sample, 2008; Harris & Cudmore, 2018). Additionally, sex of respondents (mainly females) significantly predicted accessing the registry and taking preventive actions including when the female offender lived in the community (Anderson et al., 2009; Cain et al., 2017). Being male was a predictor of agreeing with the statement “sex crimes committed by females are less serious than sex crimes committed by men” (Cain et al., 2017) and perceived community notification policies and correctional initiatives as effective methods of reducing sexual abuse (Koon-Magnin, 2015). Females had more positive views about the success potential of GPS monitoring of ICSO (Button et al., 2013), yielded higher levels of punitiveness (King, 2019), had increased risk perception of individuals registered for sexual offenses (Socia & Harris, 2016), were more likely to support Megan’s Law and were less likely to think that ICSO could be rehabilitated (Redlich, 2001), were more likely to get ICSO information from an alternative source (other than the registry), and increased the odds of checking the registry for safety reasons but decreased the odds of checking the registry for personal interest and curiosity (Sample et al., 2011). Finally, gender moderated attitudes toward the social network ban, that is, females showed more sensitivity to the “sex offender” label than males (Harris & Socia, 2016). Conversely, men were more likely to endorse more severe punishments for those accessing child sexual abuse material (Mears et al., 2008) and were more likely to report a lack of interest in accessing the registry (Sample et al., 2011).

#### Age

Six out of fifteen studies (40%) found age was associated with different variables. An association was found between age and knowing about and use of the registry (frequently), feeling safer, perceived current laws to be fair, to be less tolerant of a ICSO living in their neighborhood, and believed the current disclosure schemes are useful in reducing child sexual offenses (Anderson & Sample, 2008; Harris & Cudmore, 2018; Zgoba & Cowan, 2020). Moreover, checking the registry decreased with age and predicted both women and male access (Anderson et al., 2009). Being younger than 50 years old predicted taking preventive action if a female committed sex offenses lived in the community but being older than 50 predicted believing that female sex crimes are less serious than sex crimes committed by men (Cain et al., 2017). Belonging to the 30 to 59 age group was a significant predictor of using the registry, and the 19 to 59 age range predicted the number of times accessing the registry (Harris & Cudmore, 2018). Finally, age was a predictor of lack of interest for not using the registry (Sample et al., 2011).

#### Race

Seven out of fourteen studies (50%) found that being Caucasian correlated with knowing about the registry, accessing the registry (Anderson & Sample, 2008; Harris & Cudmore, 2018), perceiving current laws to be fair, and mandatory treatment to be beneficial to reduce sexual abuse (Zgoba & Cowan, 2020). Additionally, Caucasian respondents were less likely to hold extremely positive views about GPS monitoring (Button et al., 2013), disagree with the statement “female sex crimes are less serious than sex crimes committed by men” (Cain et al., 2017), more likely to use the registry (Harris & Cudmore, 2018), support tougher sanctions for those who access child sexual abuse material (Mears et al., 2008), and get ICSO information from an alternative source (Sample et al., 2011).

#### Education

Ten out of fifteen studies (67%) reported that education correlated with knowing about and accessing the registry (Anderson & Sample, 2008). Moreover, less educated respondents were less likely to check the registry and take preventive action (Anderson et al., 2009), supported all community policies and more severe ones (Comartin et al., 2009), and supported tougher sanctions for individuals who accessed child sexual abuse material (Mears et al., 2008). Conversely, higher education predicted extremely negative views about GPS monitoring of ICSO (Button et al., 2013), taking preventive action if a female offender lived in the community, (strongly) disagreed with the belief that female sex crimes are less serious than sex crimes committed by males (Cain et al., 2017), and decreased adherence to misconceptions about sexual offenses (King, 2019). Those who received some college education were more likely to report a lack of interest in accessing the registry (Sample et al., 2011), and a decrease in the risk perception of ICSO (Socia & Harris, 2016). Finally, misinformation was higher among those with lower education (Kernsmith et al., 2016).

#### Income

Six out of ten studies (60%) showed that income correlated with knowing about and accessing the registry, feeling safer (Anderson & Sample, 2008), and negatively correlated with supporting offender policies even severe ones (i.e., life in prison and castration) (Comartin et al., 2009). Additionally, those with higher income were less likely to hold extremely positive views on GPS monitoring of ICSO (Button et al., 2013) and more likely to report a lack of interest in accessing the registry, to check the registry because of personal interest and curiosity (Sample et al., 2011), and to be aware of local ICSO living in the area (Burchfield, 2012). Those with lower income were more likely to support tougher sanctions for those who accessed child sexual abuse material (Mears et al., 2008).

#### Living Area

Four out of five studies (80%) found that residing in an urban area correlated with accessing the registry (Anderson & Sample, 2008; Harris & Cudmore, 2018), and it was a predictor of accessing the registry (particularly for women and men living in the city) and taking preventive action by female respondents (Anderson et al., 2009). Those living in rural areas (strongly) agreed with the statement “female sex crimes are less serious than sex crimes committed by men” (Cain et al., 2017).

#### Marital Status

Five out of nine studies (55%) reported that marital status correlated with knowing about, accessing, and the number of times the registry was accessed (Anderson & Sample, 2008; Harris & Cudmore, 2018). Additionally, being married was a significant positive predictor of “neither agree nor disagree” on the belief that female crimes are less serious (Cain et al., 2017), and being more worried about a child being sexually abused by a stranger (Craun & Theriot, 2009). Moreover, those who were not married were slightly more likely to say they do not know if they would take preventive action (Cain et al., 2017), and those divorced/separated were more likely to check the registry for safety reasons, but married respondents were less likely to check the registry for personal interest and curiosity (Sample et al., 2011).

#### Other Variables

*Parental status* was significant in 9 studies (out of 15, 60%). Having children correlated with knowing about and accessing the registry (several times), feeling safer and taking preventive actions (Anderson & Sample, 2008; Harris & Cudmore, 2018), supporting policies even the more severe ones (i.e., life in prison and castration) (Comartin et al., 2009), and perceiving current laws to be fair (Zgoba & Cowan, 2020). Parents were more likely to support residence restrictions (Mancini et al., 2010) even if they are ineffective (Budd & Mancini, 2016), take preventive action if a female who committed sex offenses lived in community (Cain et al., 2017), and had greater fear (Manchak & Fisher, 2019).

*Political ideology* was examined in seven studies, but five studies (71%) found significant associations with using the registry and using it frequently (more than five times) (Harris & Cudmore, 2018). Moreover, being conservative predicted greater support of policies not based on evidence (Campbell & Newheiser, 2019), checking the registry frequently (Harris & Cudmore, 2018), and higher estimates of risk of individuals registered for sexual offenses (Socia & Harris, 2016). Finally, conservative beliefs moderated the relationship between knowledge and attitudes (Rosselli & Jeglic, 2017). Liberals perceived the registry as useful to police but unhelpful for offenders or in reducing nonsexual offenses. In addition, liberals were against residence restrictions for ICSO and were more accurate on their knowledge of who should be eligible for the registry (Jung et al., 2018).

*Victimization* was included in eight studies, and four studies (50%) reported that being victim of property crime had greater odds of holding extremely negative views about GPS monitoring of ICSO (Button et al., 2013), being victim of violent crime, and worrying of victimization increased the odds of misperception of ICSO risk (Craun & Theriot, 2009). However, neither direct nor indirect victimization had an effect on punitiveness or misconceptions of ICSO (King, 2019). Finally, victimization (especially female and older) was significantly related to fear but not for supporting community notification or severe sanctions in the mediation model (Kernsmith et al., 2016). One noteworthy finding was that victims of sexual abuse not only were more likely to have positive attitudes toward ICSO than nonvictims but also reported lower levels of support for registration and notification laws as compared with nonvictims (Spoo et al., 2018).

*Fear* was examined in two studies, and both found higher fear of ICSO significantly correlated with supporting ICSO policies even severe ones (Comartin et al., 2009; Kernsmith et al., 2016). Moreover, those with higher fear were more likely to support these policies (Comartin et al., 2009).

*Religion* was examined in three studies, and two studies (67%) found a significant association. Religion was significantly associated with using the registry more than five times (Harris & Cudmore, 2018). Additionally, Catholics were more likely to support residence restrictions even if ineffective (Budd & Mancini, 2016).

*Neighborhood characteristics* were included in three studies, and all found significant relationships with some characteristics. For instance, respondents who lived in neighborhoods with greater amounts of social capital, and with a higher rate of registered ICSO predicted extremely negative views about GPS monitoring of ICSO. Conversely, respondents living in neighborhoods with greater proportions of vulnerable populations and lower levels of incivility had greater odds of holding extremely positive views on GPS monitoring (Button et al., 2013). Higher awareness of ICSO in the neighborhood predicted higher misperception of ICSO risk (Craun & Theriot, 2009), but knowledge of community crime, number of address changes made by the offender, number of offenders within 0.1 mile of the respondent, and Hispanic immigrants predicted awareness of registered ICSO in the neighborhood (Craun, 2010). An increased perception of local informal social control (i.e., residents willing to intervene in local problems) decreased the likelihood of awareness of local ICSO, and residents who had lived in the neighborhood for a shorter length of time and who perceived informal social control were more likely to perceive their neighbors as likely to report a local ICSO (Burchfield, 2012).

*Perceptions, knowledge, and attitudes toward ICSO and policies* were examined across 11 studies, which found the following: greater misconceptions (i.e., stranger danger, sex offense rates are up, almost all recidivate, treatment is a waste, and the registry is effective) predicted supporting residence restrictions even it is ineffective (Budd & Mancini, 2016), punitiveness (King, 2019), reduced concern about unintended effects of the registry (i.e., harassment) (Mancini, 2014), increased risk perception of individuals registered for sexual offenses (Socia & Harris, 2016), and supported community notification and severe sanctions (Kernsmith et al., 2016). Other beliefs, such as research, would not change an individual’s views about the registry and notification and supporting increased funding for the registry predicted increased risk perception (Socia & Harris, 2016). Those with higher knowledge of ICSO were less likely to support community notification and residence restriction laws (Spoo et al., 2018). Additionally, residents who are familiar with ICSO laws, who generally think ICSO deserve the laws, and those with higher income were more likely to be aware of the presence of local ICSO, whereas residents who believe that ICSO should keep their status a secret were more likely to perceive their neighbors as likely to report a local ICSO (Burchfield, 2012). Likewise, residents “very familiar” with Megan’s law, who had knowledge of community crime, the number of address changes made by an offender, and the number of offenders within 0.1 mile of the respondent, were more likely to be aware of registered ICSO in the neighborhood (Craun, 2010). Finally, negative attitudes toward ICSO correlated with higher support for the registry, and the more people believe good things happen to good people, the more they believed that the registry protected the public and prevented sexual and nonsexual offenses (Jung et al., 2018). Greater support for ICSO policies was associated with more social distancing, higher perception of offenders’ riskiness, offender criminal history, and victim age (Manchak & Fisher, 2019).

## Discussion

Public policies are often strongly influenced by public opinions (Burstein, 2003; CSOM, 2010). Public opinions, therefore, can have significant implications on policymaker decisions (Levenson et al., 2007). It is vital to understand public perceptions of ICSO policies to inform the design—and promotion—of alternative strategies, along with informing rehabilitation and reintegration interventions. In turn, the current systematic review and meta-analysis set out to synthesize the evidence on the publics’ perceptions toward ICSO community management policies, examined whether the public hold misconceptions about these laws, and identify what factors impact on these attitudes. However, these results must be interpreted with caution given the low quality of the quantitative studies included. In this section, the findings are discussed as posited in the “Methods” section.

### Meta-Analysis

Overall, findings indicated significantly high heterogeneity across the studies measuring different attitudes and perceptions. This is consistent with previous studies that have found substantial variation in public opinion (see Mancini, 2014) along with inconsistency in the measurement of attitudes toward ICSO (Harper et al., 2017). Findings from the meta-analysis indicate that respondents showed high levels of support toward these policies, particularly notification, registration, residence restrictions, and controlling movement using GPS. Even the more severe policies, such as chemical castration and life/long prison, had a considerable support. While findings indicated the public support such as policies, it was interesting to find that they had less belief in the effectiveness of these policies in reducing recidivism and protecting children. This suggests the public could be receptive of alternative options as levels of support for the policies are not on par with their perceived effectiveness. If policymakers introduce alternative methods, for example, the Circles of Support and Accountability program (Clarke et al., 2017), education on the effectiveness would be required, potentially eliciting support. Also providing evidence on the effectiveness could assist in allaying fear among the public on the new policy (Clarke et al., 2017). One idea for educating the public on new policies (and even current policies) is to provide the public with a cost–benefit analysis, outlining both the tangible and intangible costs to ensure they feel well-informed about the policy and can take any appropriate action should they wish to.

Perceptions of the most effective policy was GPS, followed by notification. Registration alone was not perceived to be effective. Perhaps this is the reason why most did not access the registry or took preventive action even after accessing the registry. It was not surprising to find that perceptions of safety (for themselves, the family, and the community) were similar to perceptions of effectiveness. The majority of respondents were also not fully aware or underestimated the collateral consequences of these laws. This finding is problematic when evidence shows registered ICSO are harassed in person, by phone and mail and physically assaulted, and afraid of their own safety (see Mancini, 2014). Further to these findings, the current study found new sources of variability such as sample type; compared with students, community members were more familiar with the policies, believed the policies were more effective, and were slightly less aware of the negative consequences of registration. Future research into potential sources of variability on public perceptions of these policies is required. Doing so might lead to a revaluation and modification of how such policies are promoted, including the potential of a more tailored approach when educating the public (e.g., community members vs. college campuses).

Of concern was the low quality of quantitative studies, particularly that none of these studies reached the minimum threshold as defined by the Q-SSP. One explanation might be the problematic conceptualization and operationalization of the key construct. Attitudes are individual evaluative dispositions that have a complex structure that involves beliefs, emotions, and behavioral responses (Ajzen & Fishbein, 2005, Hamilton & Johnson, 2020). Research that examines attitudes toward individuals who sexually offend generally has inconsistent measurements with scales that do not include these three key components. They are also more likely to focus on perceptions or stereotypical views rather than an evaluation of the topic (Harper et al., 2017). Moreover, many measures in the literature have been inadequately validated, show an inconsistent factor structure, or are based on nonstandardized and nonvalidated measures (Harper et al., 2017; Shackley et al., 2014).

Although information about citizens’ stereotypes and knowledge about sex offender policies is useful, the challenge for future studies is to consistently implement validated and comprehensive scales of attitudes like the Attitudes to Sexual Offenders (ATS) (and its short version ATS-21) and the Perceptions of Sex Offenders (Harper & Hogue, 2015; Harper et al., 2017). Harper et al. also suggest the inclusion of indirect assessment procedures, for example, the Single Target Implicit Association Test to overcome frequent problems of direct self-report assessments (e.g., social desirability) (Harper & Bartels, 2016; Harper et al., 2017). However, the application of indirect assessments must also be carefully implemented given its quality as a psychological tool has also been criticized for its reliability and validity in other areas of research such as racial prejudice (Blanton et al., 2009; Mitchell & Tetlock, 2017).

A second explanation for the low quality of studies is that the overall quality of the reviewed studies might have been underrated. As previously stated, the Q-SSP uses several responses. The response “not stated clearly” penalized studies with the same score (0) as those studies reviewed as not applying the criteria (“No”) in 13/20 items. The “not stated clearly” response was applied when the research provided “limited or unclear information” (Protogerou & Hagger, 2019) rather than the authors not applying the criteria. Limited information might also be related to journal publication guidelines (i.e., word limit, conflict of interests declaration not demanded, etc.) rather than the authors’ omissions. In the current results, almost 20% of responses were coded “not stated clearly.”

A final explanation for the low quality of studies might also reflect the different methodological practices within psychology and criminology. Although the Q-SSP is developed by psychologists, several of the studies reviewed in this research were conducted by criminologists and submitted to criminological or nonpsychological journals (*Criminal Justice Policy Review*, *Police Practice and Research*, *Journal of Sociology and Welfare*, etc.). Arguably, the standards regarding methodological quality (e.g., justification of sample, the validity of instruments, methods to deal with attrition, debriefing of participants, etc.) and its reporting might be different and/or not institutionalized as common practice.

### Knowledge and Misconceptions About Community Management Policies for ICSO

Well, under half of the respondents did not believe the myth that many sex offenses are committed by strangers. This was a positive finding, particularly when community notification and registration policies are based on the belief that these crimes are committed by strangers (Winick, 2003). Respondents also seemed to have positive views on the treatment for ICSO, consistent with the literature on treatment. For example, McKillop et al. (2022) found ICSO who completed preparatory programs, rehabilitation programs, and reintegration programs were less likely to reoffend compared with those offenders who partially completed or did not complete programs, when controlling for various factors such as risk and age. It was also pleasing to find that respondents acknowledged that the media exaggerated the level of risk of ICSO and the risk of becoming a sexual abuse victim. This is important when myths about ICSO can be perpetuated by the media (Zatkin et al., 2021). In fact, Zatkin et al. (2021) warn of the cyclical relationship, in which the media perpetuates myths, with policy being driven by myths, and then policy leading to media reporting. However, results from the current meta-analysis highlight that the public believe policymakers should more so rely on the effectiveness of the laws rather than what the public wants. These findings suggest policymakers can place less emphasis on citizens’ wants (and possible misconceptions) with a stronger focus on evidence-based policies.

Aside from these findings, there appeared to be several prevalent misconceptions and lacking knowledge. For example, despite respondents acknowledging most offenders were not strangers, it was contradictory to find that most agreed that one of the best ways to protect children from sexual abuse was teaching children about stranger danger (King, 2019). Findings also revealed misconceptions such as the increase of sex crime rates, most registered ICSO were pedophiles or sexual predators, and high recidivism rates among ICSO. However, evidence shows that levels of reoffending in follow-up periods of 5 to 15 years is roughly 15% to 30% (Hanson & Morton-Bourgon, 2005). Additionally, respondents believed that registration and notification were constitutional, and they had a lack of knowledge on the level of risk and typically suggested they would place all ICSO on the registry. Interestingly, half or more respondents suggested they supported the laws even if research showed otherwise. Given misconceptions held by the public, these findings suggest further education among the public is required. Education could be guided by both lawmakers and researchers (Koon-Magnin, 2015; Sample et al., 2011) and precede the implementation of evidence-based legislation (Koon-Magnin, 2015). As aforementioned, adopting a more tailored and targeted approach when educating the public could be beneficial, which particularly considers some of the following factors.

### Factors Contributing to Attitudes and Perceptions of Community Management Policies for ICSO

A variety of factors were found to be associated with perceptions of the policies: sex, age, race, education, income, living area, and marital status. Other variables, which were less commonly examined in the studies, included parental status, political ideology, victimization, fear, religion, neighborhood, and views and knowledge on ICSO and the policies. The findings suggest factors contributing to perceptions of community management policies for ICSOs are complex and diverse. Key findings suggest that being female, Caucasian, less educated, of lower income, having children, having higher fear, and holding greater misconceptions were related to higher levels of punitiveness, perceived current laws to be fair, or supported all policies, including the more severe ones. These findings, broadly, could be explained by the social vulnerability perspective. Although this theoretical framework is often applied in the geographical field, there is merit in exploring its application in the current context. This perspective suggests an individual’s potential exposure to risk and recovery success is shaped by a convergence of economic, social, and political factors (Wisner et al., 2004). In this instance, individuals who may, for example, be lacking resources or have children might perceive greater vulnerability and risk, thus have more fear of ICSOs and support around punitiveness.

Other key findings include being female, Caucasian, belonging to the 30 to 59 years age group, having higher education, having lower income, residing in an urban area, being divorced/separated, having children, and being conservative were more likely to use the registry, and often were more likely to know about the registry. Other key findings include being male, Caucasian, having higher education, having higher income, and being the victim of property crime were associated with more negative views about GPS monitoring of ICSO. Being male, having some college education, not being conservative, not being the victim of violent crime, having less awareness of ICSO in the neighborhood (neighborhood characteristics), and holding less misconceptions were associated with lower risk perceptions of ICSO. Finally, being female (including residing in an urban area), having higher education, and having children were associated with taking preventive action. It was particularly interesting to find that, overall, despite being female was associated with higher levels of punitiveness, more likely to know about and use the registry, higher levels of risk perceptions, and higher levels of taking preventive action, being male was a predictor of perceiving community notification policies and correctional initiatives as effective methods of reducing sexual abuse. This finding suggests that although females are more engaged with the community management policies, they may be more skeptical of them. Further research is required to unpack this gendered difference.

Another particularly noteworthy finding was that victims of sexual abuse were more likely to have positive attitudes toward ICSO and reported lower levels of support for registration and notification as compared with nonvictims. Spoo et al. (2018) argued that one possible explanation is the nature of the victim’s relationship to the perpetrator. As victims are often related to or acquainted with the perpetrator, these victims have a more nuanced relationship with the perpetrator compared with nonvictims who may rely on media portrayals of monsters (Spoo et al., 2018). In addition, compared with nonvictims, victims may have seen the collateral consequences of the laws firsthand on the people they are acquainted with or related to, which could explain the lower levels of support for the policies. Finally, although most studies focused on predictors and correlations, only two studies explored mediators and two studies examined moderators. Future research could focus on the role of mediators and moderators, and the complex interactions, to both better understand the influence of the perceptions and to test the aforementioned social vulnerability perspective.

The current study utilized studies that included diversity across sex, age, race, education, income, living area, marital status, and other variables. Although it is important that the researchers had diversity in these samples, they acknowledge that specific findings are not applicable to all due to the uniqueness of individuals and groups. In addition, it is crucial to note that only a few studies explored race, and of these studies, race what typically treated as a dichotomous variable (e.g., 1 = Caucasian and 0 = non-White/other/multiple racial/ethnic groups). It is important that future research explores more diverse perspectives in relation to race, particularly when research has found that maintaining racial variation (e.g., “Black,” “Hawaiian,” and “Hispanic”) can result in trends that are not uncovered when groups are collapsed into only one “non-White” category (Shaw & Lee, 2019). Shaw and Lee (2019) recommended that being thoughtful in deciding how to examine race and argue, it is likely to lead to a greater understanding and more nuanced findings.

### Limitations

Some limitations to this review need to be considered. First, there is an important gap in the literature examining the views of the public toward community management laws in other countries. Despite this review including studies from different countries, a large proportion of studies came from the United States. One reason for this finding might be the cultural interest in the management of ICSO, with these policies becoming very popular throughout the United States (Socia, 2012) and the proliferation of policies that the United States saw since the 1990s (Terry, 2015). Due to most of the studies coming from the United States, the findings need to be interpreted with caution and cannot be generalized to other countries (especially to those countries where the register is not open to the public). Second, this review focused mainly on the notification and register polices; there were a limited number of studies on other policies such as residence restrictions, GPS, and castration.

A third limitation was that the studies used a cross-sectional design, so the authors could not evaluate whether the perceptions varied over time. Although research suggests attitudes toward these policies are dynamic over time (Mears et al., 2008; see also Harper et al., 2017), future studies should explore this change and the possible factors affecting this change. Finally, the studies did not meet the score for acceptable quality. Low quality might be, in part, associated with the underrating of studies with information that is not clearly reported. There is a need to improve the reporting of some methodological aspects in the measurement of attitudes toward community management of policies for ICSO. The quality of studies included in systematic reviews and meta-analyses is crucial in the design and implementation of evidence-based policies. To avoid using misleading information, it is key to include a standardized checklist to better understand the quality of studies, and which specific domains need more improvement. One crucial step is to improve the quality and transparency of studies, providing more valid and useful information for policymakers.

## Conclusion

Despite limitations associated with the low quality of studies, the present study adds to the existing literature by providing a systematic review and meta-analysis of the evidence on the publics’ perceptions and attitudes of community management policies for ICSO, along with whether the public hold misconceptions about these laws, and the factors that may impact these perceptions. This review provides comprehensive evidence that these policies are supported by the community, despite their lesser belief on the effectiveness of these policies in reducing recidivism and protecting children. The public perceived notification as more effective than registration and, similarly, notification resulted in higher feelings of safety than registration. The findings on perceived effectiveness and safety concerning the registry likely explain why, overall, the public neither used the registry nor took preventive actions from it. Moreover, a moderate level of misconceptions was reported. It was, however, pleasing to find that respondents acknowledged that few sex offenses are committed by strangers, along with the role of the media in exaggerating the level of risk, and held positive views of how beneficial treatment is for ICSO. However, misconceptions were still evident (e.g., high recidivism rates and most registered ICSO are pedophiles or sexual predators). Finally, there were key differences in perceptions across factors including sex, age, race, education, income, living area, and marital status, highlighting the complexity and diversity of factors associated with perceptions. Future research should explore the role of mediators and moderators, and the complex interactions, to better understand the phenomenon. Such findings could assist with the revaluation and modification of how policies are promoted. Education for the pubic, including a cost–benefit analysis, needs to precede the implementation of any evidence-based legislation, and could involve a more tailored and targeted approach that draws from the diverse factors discussed in the current study.

**Table:** 

Summary: Critical Findings
• Respondents showed high levels of support toward these policies, particularly notification, registration, residence restrictions, and controlling movement using GPS. • Despite this support, the public had less belief in the effectiveness of these policies in reducing recidivism and protecting children. • Perceptions of safety (for themselves, the family, and the community) were similar to their perceptions of effectiveness. • Most respondents were not fully aware or underestimated the collateral consequences of these laws. • Although there were some clear advancements in the publics’ misconceptions, there appeared to still be several prevalent misconceptions and lacking knowledge. • The factors associated with perceptions of community management policies for ICSO are complex and diverse. • These factors included sex, age, race, education, income, living area, and marital status. Other variables, that were less commonly examined in the studies, included parental status, political ideology, victimization, fear, religion, neighborhood, and views and knowledge on ICSO and the policies.
Summary: Implications of the Review for Practice, Policy, and Research
• Findings suggest policymakers can place less emphasis on citizens’ wants (and possible misconceptions) with a stronger focus on evidence-based policies. • If policymakers introduce alternative policies, the public will require education on the effectiveness, which may also lead to greater support and a reduction in fear. A cost–benefit analysis, outlining both the tangible and intangible costs is suggested. • Further research into potential sources of variability on public perceptions of these policies is required, potentially resulting in the revaluation and modification of how such policies are promoted, including the potential of a more tailored approach when educating the public. • The social vulnerability perspective might explain how some of the demographic factors affect the public’s attitudes. • Future research could explore the role of mediators and moderators, and the complex interactions, to both better understand the influence of the perceptions and to test the social vulnerability perspective.

## Supplemental Material

sj-docx-1-tva-10.1177_15248380231174695 – Supplemental material for Public Perceptions Toward Community Management Policies for Individuals Convicted of Sexual Offenses: A Systematic Review and Meta-Analysis Supplemental material, sj-docx-1-tva-10.1177_15248380231174695 for Public Perceptions Toward Community Management Policies for Individuals Convicted of Sexual Offenses: A Systematic Review and Meta-Analysis by Olga Sánchez de Ribera, Larissa S. Christensen, Nicolás Trajtenberg and Kirsty Hudson in Trauma, Violence, & Abuse

## Notes

1. Similar to Christensen et al. (2021), the authors selected the commencing year as 1994 because this was when the U.S. Congress passed the *Jacob Wetterling Crimes Against Children and Sexually Violent Offender Registration Act.*

2. Four studies were excluded (Beshears, 2017; Campbell & Newheiser, 2019; Koon-Magnin, 2015; Manchak & Fisher, 2019) because the focus was on the random assignment of items or even questionnaires in different geographical areas and the measurement of perceptions and the survey was a secondary aspect of the study.

## References

[bibr1-15248380231174695] Ajzen I. Fishbein M. (2005). The influence of attitudes on behavior. In Albarracín D. Johnson B. T. Zanna M. P. (eds.), The handbook of attitudes (pp. 173–221). Lawrence Erlbaum Associates Publishers.

[bibr2-15248380231174695] *Anderson A. L. Evans M. K. Sample L.L. (2009). Who accesses the sex offender registries? A look at legislative intent and citizen action in Nebraska. Criminal Justice Studies, 22(3), 313–329. 10.1080/14786010903167096

[bibr3-15248380231174695] *Anderson A. L. Sample L. L. (2008). Public awareness and action resulting from sex offender community notification laws. Criminal Justice Policy Review, 19(4), 371–396. 10.1177/0887403408316705

[bibr4-15248380231174695] Baker T. Metcalfe C. F. Berenblum T. Aviv G. Gertz M. (2015). Examining public preferences for the allocation of resources to rehabilitative versus punitive crime policies. Criminal Justice Policy Review, 26(5), 448–462. 10.1177/0887403414521462

[bibr5-15248380231174695] Barendregt J. J. Doi S. A. Lee Y. Y. Norman R. E. Vos T. (2013). Meta-analysis of prevalence. Journal of Epidemiology & Community Health, 67(11), 974–978. 10.1136/jech-2013-203104 23963506

[bibr6-15248380231174695] *Beck V. S. Clingermayer J. Ramsey R. J. Travis L. (2004). Community response to sex offenders. Journal of Psychiatry and Law, 32(2), 141–168. 10.1177/009318530403200202

[bibr7-15248380231174695] Bedarf A. (1995). Examining sex offender community notification laws. California Law Review, 83(3), 885–939. 10.2307/3480867

[bibr8-15248380231174695] *Berryessa C. M. Lively C. (2019). When a sex offender wins the lottery: Social and legal punitiveness toward sex offenders in an instance of perceived injustice. Psychology, Public Policy, and Law, 25(3), 181–195. 10.1037/law0000198.

[bibr9-15248380231174695] *Beshears M. L. (2017). Neighbors of registered sex offenders: A qualitative analysis of their lived experiences. International Journal of Law, Crime and Justice, 48, 40–49. 10.1016/j.ijlcj.2016.10.003

[bibr10-15248380231174695] Blanton H. Jaccard J. Klick J. Mellers B. Mitchell G. Tetlock P. E. (2009). Strong claims and weak evidence: Reassessing the predictive validity of the IAT. Journal of Applied Psychology, 94(3), 567–582. 10.1037/a0014665 19449998

[bibr11-15248380231174695] Borenstein M. Hedges L. V. Higgins J. P. T. Rothstein H. R. (2011). Introduction to meta-analysis. Wiley.

[bibr12-15248380231174695] *Brannon Y. N. Levenson J. S. Fortney T. Baker J. N. (2007). Attitudes about community notification: A comparison of sexual offenders and the non-offending public. Sexual abuse: A Journal of Research and Treatment, 19(4), 369–379. 10.1177/107906320701900403 17924191

[bibr13-15248380231174695] *Brown S. Deakin J. Spencer J. (2008). What people think about the management of sex offenders in the community. The Howard Journal of Criminal Justice, 47(3), 259–274. 10.1111/j.1468-2311.2008.00519.x

[bibr14-15248380231174695] *Budd K. M. Mancini C. (2016). Crime control theater: Public (mis)perceptions of the effectiveness of sex offender residence restrictions. Psychology, Public Policy, and Law, 22(4), 362–374. 10.1037/law0000083

[bibr15-15248380231174695] *Burchfield K. B. (2012) Assessing community residents’ perceptions of local registered sex offenders: Results from a pilot survey. Deviant Behavior, 33(4), 241–259. 10.1080/01639625.2011.573396

[bibr16-15248380231174695] Burstein P. (2003). The impact of public opinion on public policy: A review and an agenda. Political Research Quarterly, 56(1), 29–40. 10.1177/106591290305600103

[bibr17-15248380231174695] *Button D. M. Tewksbury R. Mustaine E. E. Payne B. K. (2013). Factors contributing to perceptions about policies regarding the electronic monitoring of sex offenders: The role of demographic characteristics, victimization experiences, and social disorganization. International Journal of Offender Therapy and Comparative Criminology, 57(1), 25–54. 10.1177/0306624X11426698 22158910

[bibr18-15248380231174695] *Cain C. M. Sample L. L. Anderson A. L. (2017). Public opinion of the application of sex offender notification laws to female sex offenders: Why it is important to examine. Criminal Justice Policy Review, 28(2), 155–175. 10.1177/0887403415572253

[bibr19-15248380231174695] *Campbell D. S. Newheiser A.-K. (2019). Must the show go on? The (in)ability of counterevidence to change attitudes toward crime control theater policies. Law and Human Behavior, 43(6), 568–584. 10.1037/lhb0000338 31380671

[bibr20-15248380231174695] *Center for Sex Offender Management (CSOM). (2010). Public awareness and attitudes about sex offender management: Findings from a national public opinion poll. U.S. Department of Justice.

[bibr21-15248380231174695] Christensen L. S. Sánchez de Ribera O. Trajtenberg N. (2021). A systematic review of professionals’ views about community management policies for individuals convicted of sexual offenses. Sexual Abuse, 34(2), 127–156. 10.1177/10790632211000369 33724102

[bibr22-15248380231174695] *Chui W. H. Cheng K. K. Yoke-chan Ong R. (2015). Attitudes of the Hong Kong Chinese public towards sex offending policies: The role of stereotypical views of sex offenders. Punishment & Society, 17(1), 94–113. 10.1177/1462474514561712

[bibr23-15248380231174695] Clarke M. Brown S. Völlm B. (2017). Circles of support and accountability for sex offenders: A systematic review of outcomes. Sexual Abuse, 29(5), 446–478. 10.1177/1079063215603691 26369806

[bibr24-15248380231174695] *Comartin E. B. Kernsmith P. D. Kernsmith R. M. (2009). Sanctions for sex offenders: Fear and public policy. Journal of Offender Rehabilitation, 48(7), 605–619. 10.1080/10509670903196066

[bibr25-15248380231174695] Connor D. P. Tewksbury R. (2017). Public and professional views of sex offender registration and notification. Criminology, Criminal Justice, Law & Society, 18(1), 1–27.

[bibr26-15248380231174695] *Craun S. W. (2010). Evaluating awareness of registered sex offenders in the neighborhood. Crime & Delinquency, 56(3), 414–435. 10.1177/0011128708317457

[bibr27-15248380231174695] *Craun S. W. Theriot M. T. (2009). Misperceptions of sex offender perpetration: Considering the impact of sex offender registration. Journal of Interpersonal Violence, 24(12), 2057–2072. 10.1177/0886260508327706 19074256

[bibr28-15248380231174695] Duwe G. Donnay W. Tewksbury R. (2008). Does residential proximity matter? A geographic analysis of sex offense recidivism. Criminal Justice and Behavior, 35(4), 484–504. 10.1177/0093854807313690

[bibr29-15248380231174695] *Garland B. Calfano B. Wodahl E. (2018). College student perceptions of notification about sex offenders on campus. Criminal Justice Policy Review, 29(3), 240–257. 10.1177/0887403416651670

[bibr30-15248380231174695] Hamilton K. Johnson B. T. (2020). 31 attitudes and persuasive communication interventions. In Hagger M. S. Cameron L. D. Hamilton K. Hankonen N. Lintunen T. (Eds.), The handbook of behavior change (pp. 445–460). Cambridge University Press.

[bibr31-15248380231174695] Hanson R. K. Morton-Bourgon K. E. (2005). The characteristics of persistent sexual offenders: A meta-analysis of recidivism studies. Journal of Consulting and Clinical Psychology, 73(6), 1154–1163. 10.4324/9781351161565-5 16392988

[bibr32-15248380231174695] Harper C. A. Bartels R. M. (2016). Implicit theories and offender representativeness in judgments of sexual crime. Sexual Abuse: A Journal of Research and Treatment, 30(3), 276–295. 10.1177/1079063216658019 27369242

[bibr33-15248380231174695] Harper C. A. Hogue T. E. (2015). Measuring public perceptions of sex offenders: Reimagining the Community Attitudes Toward Sex Offenders (CATSO) scale. Psychology, Crime & Law, 21(5), 452–470. 10.1080/1068316X.2014.989170

[bibr34-15248380231174695] Harper C. A. Hogue T. E. Bartels R. M. (2017). Attitudes towards sexual offenders: What do we know, and why are they important? Aggression & Violent Behavior, 34, 201–2013. 10.1016/j.avb.2017.01.011

[bibr35-15248380231174695] *Harris A. J. Cudmore R. (2018). Community experience with public sex offender registries in the United States: A national survey. Criminal Justice Policy Review, 29(3), 258–279. 10.1177/0887403415627195

[bibr36-15248380231174695] *Harris A. J. Socia K. M. (2016). What’s in a name? Evaluating the effects of the “sex offender” label on public opinions and beliefs. Sexual Abuse, 28(7), 660–678. 10.1177/1079063214564391 25542837

[bibr37-15248380231174695] Higgins C. Ireland C. A. (2009). Attitudes towards male and female sex offenders: A comparison of forensic staff, prison officers and the general public in Northern Ireland. British Journal of Forensic Practice, 11(1), 14–19. 10.1108/14636646200900004

[bibr38-15248380231174695] Higgins J. Thompson S. (2002). Quantifying heterogeneity in meta-analysis. Statistics in Medicine, 21(11), 1539–1558. 10.1002/sim.1186 12111919

[bibr39-15248380231174695] *Jung S. Allison M. Martin E. (2018). Perspectives of Americans and Canadians on the use and function of sex offender registries. International Journal of Law, Crime and Justice, 52, 106–117. 10.1016/j.ijlcj.2017.10.003.

[bibr40-15248380231174695] *Kernsmith P. Comartin E. Kernsmith R. (2016). Fear and misinformation as predictors of support for sex offender management policies. Journal of Sociology Social Welfare, 43(2), 39–66.

[bibr41-15248380231174695] *Kernsmith P. D. Craun S. W. Foster J. (2009). Public attitudes toward sexual offenders and sex offender registration. Journal of Child Sexual Abuse, 18(3), 290–301. 10.1080/10538710902901663 19856734

[bibr42-15248380231174695] *King L. L. (2019). Perceptions about sexual offenses: Misconceptions, punitiveness, and public sentiment. Criminal Justice Policy Review, 30(2), 254–273. 10.1177/0887403416660150

[bibr43-15248380231174695] *Klein J. L. Cooper D. T. (2019). Punitive attitudes toward sex offenders: Do moral panics cause community members to be more punitive? Criminal Justice Policy Review, 30(6), 948–968. 10.1177/0887403418767251

[bibr44-15248380231174695] *Koon-Magnin S. (2015). Perceptions of and support for sex offender policies: Testing Levenson, Brannon, Fortney, and Baker’s findings. Journal of Criminal Justice, 43(1), 80–88. 10.1016/j.jcrimjus.2014.12.007

[bibr45-15248380231174695] *Levenson J. Brannon Y. N. Fortney T. Baker J. N. (2007). Public perceptions about sex offenders and community protection policies. Analyses of Social Issues and Public Policy, 7(1), 137–161. 10.1111/j.1530-2415.2007.00119.x

[bibr46-15248380231174695] *Lieb R. Nunlist C. (2008). Community notification as viewed by Washington’s Citizens: A 10-year follow-up (Document No. 08-03-1101). Washington State Institute for Public Policy.

[bibr47-15248380231174695] *Manchak S. M. Fisher L. R. (2019). An examination of multiple factors influencing support for sex offender policy. Criminal Justice Policy Review, 30(6), 925–947. 10.1177/0887403417715684

[bibr48-15248380231174695] *Mancini C. (2014). Examining factors that predict public concern about the collateral consequences of sex crime policy. Criminal Justice Policy Review, 25(4), 450–475. 10.1177/0887403412473475

[bibr49-15248380231174695] *Mancini C. Shields R. T. Mears D. P. Beaver K. M. (2010). Sex offender residence restriction laws: Parental perceptions and public policy. Journal of Criminal Justice, 38(5), 1022–1030. 10.1016/j.jcrimjus.2010.07.004

[bibr50-15248380231174695] McKillop N. Hine L. Rayment-McHugh S. Prenzler T. Christensen L. S. Belton E. (2022). Effectiveness of sexual offender treatment and reintegration programs: Does program composition and sequencing matter? Journal of Criminology, 55(2), 180–201. 10.1177/26338076221079046

[bibr51-15248380231174695] *Mears D. P. Mancini C. Gertz M. Bratton J. (2008). Sex crimes, children, and pornography: Public views and public policy. Crime & Delinquency, 54(4), 532–559. 10.1177/0011128707308160

[bibr52-15248380231174695] Meloy M. Curtis K. Boatwright J. (2013). The sponsors of sex offender bills speak up: Policy makers’ perceptions of sex offenders, sex crimes, and sex offender legislation. Criminal Justice and Behavior, 40(4), 438–452. 10.1177/0093854812455740

[bibr53-15248380231174695] Mercado C. C. Alvarez S. Levenson J. (2008). The impact of specialized sex offender legislation on community reentry. Sexual Abuse, 20(2), 188–205. 10.1177/1079063208317540 18490481

[bibr54-15248380231174695] Mitchell G. Tetlock P. E. (2017). Popularity as a poor proxy for utility: The case of implicit prejudice. In Lilienfeld S. Waldman I. D. (Eds.), Psychological science under scrutiny: Recent challenges and proposed solutions (pp. 164–195). John Wiley and Sons.

[bibr55-15248380231174695] Moher D. Liberati A. Tetzlaff J. Altman D. G. , & The PRISMA Group (2009). Preferred reporting items for systematic reviews and meta-analyses: The PRISMA statement. Annals of Internal Medicine, 151, 264–269. 10.7326/0003-4819-151-4-200908180-00135 19622511

[bibr56-15248380231174695] Mustaine E. E. Tewksbury R. Stengel K. M. (2006). Social disorganization and residential locations of registered sex offenders: Is this a collateral consequence? Deviant Behavior, 27(3), 329–350. 10.1080/01639620600605606

[bibr57-15248380231174695] *Phillips D. (1998). Community notification as viewed by Washington’s citizens. Washington State Institute for Public Policy.

[bibr58-15248380231174695] Protogerou C. Hagger M. S. (2020). A checklist to assess the quality of survey studies in psychology. Methods in Psychology, 3, 1–14. 10.1016/j.metip.2020.100031

[bibr59-15248380231174695] Quinn J. F. Forsyth C. J. Mullen-Quinn C. (2004). Societal reaction to sex offenders: A review of the origins and results of the myths surrounding their crimes and treatment amenability. Deviant Behavior, 25(3), 215–232. 10.1080/01639620490431147

[bibr60-15248380231174695] *Redlich A. (2001). Community notification: Perceptions of its effectiveness in preventing child sexual abuse. Journal of Child Sexual Abuse, 10(3), 91–116. 10.1300/J070v10n03_06 17522002

[bibr61-15248380231174695] *Rosselli M. K. Jeglic E. L. (2017). Factors impacting upon attitudes toward sex offenders: The role of conservatism and knowledge. Psychiatry, Psychology and Law, 24(4), 496–515. 10.1080/13218719.2016.1254562 PMC6818402 31983970

[bibr62-15248380231174695] *Sample L. L. Evans M. K. Anderson A. L. (2011). Sex offender community notification laws: Are their effects symbolic or instrumental in nature? Criminal Justice Policy Review, 22(1), 27–49. 10.1177/0887403410373698

[bibr63-15248380231174695] *Schiavone S. K. Jeglic E. L. (2009). Public perception of sex offender social policies and the impact on sex offenders. International Journal of Offender Therapy and Comparative Criminology, 53(6), 679–695. 10.1177/0306624X08323454 18728128

[bibr64-15248380231174695] *Shackley M. Weiner C. Day A. Willis G. M. (2014). Assessment of public attitudes towards sex offenders in an Australian population. Psychology, Crime & Law, 20(6), 553–572. 10.1080/1068316X.2013.793772

[bibr65-15248380231174695] Shaw J. Lee H. (2019). Race and the criminal justice system response to sexual assault: A systematic review. American Journal of Community Psychology, 64(1–2), 256–278. 10.1002/ajcp.12334 PMC6796211 31059130

[bibr66-15248380231174695] Socia K. M. (2012). The efficacy of county-level sex offender residence restrictions in New York. Crime & Delinquency, 58(4), 612–642. 10.1177/0011128712441694

[bibr67-15248380231174695] *Socia K. M. Harris A. J. (2016). Evaluating public perceptions of the risk presented by registered sex offenders: Evidence of crime control theater? Psychology, Public Policy, and Law, 22(4), 375–385. 10.1037/law0000081

[bibr68-15248380231174695] *Spoo S. Kaylor L. E. Schaaf S. Rosselli M. Laake A. Johnson C. Jeglic E. L. (2018). Victims’ attitudes toward sex offenders and sex offender legislation. International Journal of Offender Therapy and Comparative Criminology, 62(11), 3385–3407. 10.1177/0306624X17740537 29110538

[bibr69-15248380231174695] *Taylor S. C. (2017). Community perceptions of a public sex offender registry introduced in Western Australia. Police Practice and Research, 18(3), 275–290. 10.1080/15614263.2017.1291572

[bibr70-15248380231174695] Terry K. J. (2015). Sex offender laws in the United States: Smart policy or disproportionate sanctions? International Journal of Comparative and Applied Criminal Justice, 39(2), 113–127. 10.1080/01924036.2014.973048

[bibr71-15248380231174695] Tewksbury R. Lees M. (2006). Perceptions of sex offender registration: Collateral consequences and community experiences. Sociological Spectrum, 26(3), 309–334. 10.1080/02732170500524246

[bibr72-15248380231174695] Tewksbury R. Mustaine E. (2009). Stress and collateral consequences for registered sex offenders. Journal of Public Management and Social Policy, 15(2), 215–239. 10.1002/bsl.878

[bibr73-15248380231174695] Wallace B. C. Dahabreh I. J. Trikalinos T. A. Lau J. Trow P. Schmid C. H. (2012). Closing the gap between methodologists and end-users: R as a computational back-end. Journal of Statistical Software, 49(5), 2–13.

[bibr74-15248380231174695] Winick B. J. (2003). A therapeutic jurisprudence analysis of sex offender registration and community notification laws. In Winick B. J. La Fond J. Q. (Eds.), Protecting society from sexually dangerous offenders: Law, justice, and therapy (pp. 213–229). American Psychological Association.

[bibr75-15248380231174695] Wisner B. Blaikie P. Cannon T. Davis I. (2004). At risk: Natural hazards, people’s vulnerability, and disasters (2nd ed.). Routledge.

[bibr76-15248380231174695] Wright R. (2003). Sex offender registration and notification: Public attention, political emphasis, and fear. Criminology and Public Policy, 3(1), 97–104.

[bibr77-15248380231174695] Zatkin J. Sitney M. Kaufman K. (2021). The relationship between policy, media, and perceptions of sexual offenders between 2007 and 2017: A review of the literature. Trauma, Violence, & Abuse, 23(3), 953–968. 10.1177/1524838020985568 33438524

[bibr78-15248380231174695] *Zevitz R. Farkas M. (2000). Sex offender community notification: Examining the importance of neighborhood meetings. Behavioral Sciences and the Law, 18, 383–408. 10.1002/1099-0798(200003/06)18:2/3<393::aid-bsl381>3.0.co;2-o 10874295

[bibr79-15248380231174695] *Zgoba K. M. Cowan D. (2020). Sexual offense legislation across the pond: A review of community sentiment toward the United Kingdom’s implementation of Sarah’s Law. Sexual Abuse, 32(4), 476–496. 10.1177/1079063219847671 31109251

