# Supplementary material for: Public Perceptions Toward Community Management Policies for Individuals Convicted of Sexual Offenses: A Systematic Review and Meta-Analysis
Source: Trauma Violence Abuse. 2023 May 18;25(2):1248–64. doi: 10.1177/15248380231174695 (PMC10913335; doi:10.1177/15248380231174695)
Supplement: sj-docx-1-tva-10.1177_15248380231174695 – Supplemental material for Public Perceptions Toward Community Management Policies for Individuals Convicted of Sexual Offenses: A Systematic Review and Meta-Analysis [file sj-docx-1-tva-10.1177_15248380231174695.docx]

Appendix A.

Table 1.

*Descriptive characteristics of included studies*

| Author(s), year | Country | Date of data  collection | Sample size | Sample characteristics  (type, gender, age) | Sampling technique | Method | Response rate | Study quality  (Acceptable ≥75%) |
| --- | --- | --- | --- | --- | --- | --- | --- | --- |
| Anderson & Sample, 2008 | US | November 2006 to March 2007 | 1,821 | CM,  F: 50.5% M:49.5%,  25 to 44: 36.6%  45 to 64: 33.8% | RDD | Phone survey  (and comments) | 33% | Yes or No |
| Anderson et al. 2009 | US | November 2006 to March 2008 | 1,821 | CM,  F: 50.5% M:49.5%,  26 to 44: 36.6%  45 to 64: 33.8% | RDD | Phone survey  (and comments) | 33% |  |
| Beck et al. 2004 | US | NR | 236 | CM,  F: 66.1% M:33.9%,  median: 45 | Strata purposive | Hamilton County survey (2002 survey of Crime Perceptions and Crime Protection) | 42% for notified group  30% for non-notified group |  |
| Berryessa & Lively, 2019 | US | June 2015 | Study 1: 1,005,  S2: 370,  S3: 400 | CM,  Study 1: F:43.78% M:56.22%,  S2: F:45.68% M:54.32%,  S3: F:42% M:58.00%,  Study 1, mean: 33.47 (9.72)  S2: 34.67(11.57), S3: 34.37(11.13) | Random | Online survey  with vignettes | NR |  |
| Beshears 2017 | US | NR | 20 | CM,  NR,  NR | Convince | Face to face interview | NA |  |
| Brannon et al. 2007 | US | July-August 2005 | 193 | CM,  F:57% M:43%,  mean:39 | Convenience | Questionnaire | 98% |  |
| Brown et al. 2008 | UK | February-July 2005 | 979 | CM  Mixed (% NR)  Mainly within the range of 56+ | Convenience | Online/qualitative and postal/quantitative questionnaires,  Meetings with community groups | 15% |  |
| Budd & Mancini, 2016 | US | February-March 2010 | 1005 | CM,  F:59.9% M:40.1%, | RDD | Survey | NR |  |
| Burchfield, 2012 | US | NR | 95 | CM,  NR,  46.87 years (18.85) | Census block groups, 22 blocks randomly selected | Door-to-door survey | 69% |  |
| Button et al. 2013 | US | During the spring of 2007 | 746 | CM,  F:68.8% M:31.2%,  48.9 years (16.6) | Stratified and random telephone numbers | Telephone survey | NR |  |
| Cain et al. 2017 | US | June 14, 2012-September 12, 2012 | 954 | CM,  F:50.2% M:47.8%,  19 to 49: 52.3%,  50 and older: 45.0% | Random | 2012 Nebraska Annual Social Indicators Survey | 27.2% |  |
| Campbell & Newheiser, 2019 | US | NR | Study 1: 298  Study 2: 147  Study 3: 552 | CM,  F:48%, 40%, 43% M:52%, 60%, 57%  M=35.6(SD=10.5) 35.03 (SD= 9.54)  35.86 years (*SD* _ 11.46), | Random | Experiments  Questionnaire, with an open-ended question for study 2 | Study 1: 95.5%  Study 2: 95%  Study 3: 97% |  |
| CSOM, 2010 | US | February-March 2010 | 1,005 | CM,  NR  NR | Random | Telephone survey | NR | No |
| Chui et al. 2015 | Hong Kong | In 2011 | 202 | CM,  F: 70.8% M:29.3%  Median: 42.0. | Random | Telephone survey | 51 |  |
| Comartin et al. 2009 | US | NR | 703 | CM,  F:62.2 M:37.8,  *M=*51.44(*SD*=16.16) | RDD | Telephone surveys | 46 |  |
| Craun & Theriot, 2009 | US | NR | 631 | CM,  F: 66.2% M:33.8%,  *M*=48.0 years old (Median = 47) | Randomly selected residential addresses in a single county | Mail survey | 45% |  |
| Craun, 2010 | US | NR | 631 | CM,  F: 65.7 M:34.2,  Median: 48.7 | Random | Mail survey | 45% |  |
| Garland et al. 2018 | US | NR | 1,048 | Students,  F: 60.1% M:39.9%  NR | Random classes | Online survey | NR |  |
| Harris & Cudmore, 2018 | US | March-April 2015 | 1,000 | CM,  F:52.0 M:48.0,  60+: 30.4%,  30-44: 29.5%,  45-59: 23.9%,  19-29: 16.2% | Two stage: non-probability over sample from internet panel. Representative sample using propensity score matching | Online survey | NR |  |
| Harris & Socia, 2016 | US | April 2014 | 1,000 | CM,  F:53.6 M:46.4,  Median: 48.0 | Representativeness of non-probability Internet samples | Online survey. Experiment | NR |  |
| Jung et al. 2018 | Canada  US | NR | 844  (Students: 207  CM: 637) | CM, Students  F:58.5% M:40.6%,  18–25 years:36.6%, 26–35 years: 27.6%, 36–45 years: 15.3%... | NR | Online survey | NR |  |
| Kernsmith et al. 2009 | US | NR | 733 | CM,  F:67.3% M:32.7%,  50.5 years (SD = 16.83) | RDD | Telephone survey | 44% |  |
| Kernsmith et al. 2016 | US | NR | 703 | CM,  F:62.2% M:37.8%,  51.44 years, (SD = 16.16) | RDD | Telephone survey | 46% |  |
| King, 2019 | US | NR | 174 | CM,  F: 55.5% M:44.5%,  M = 49.08 (SD = 15.99) | Multistage cluster sampling | Mail and online survey | NR |  |
| Klein & Cooper, 2019 | US | During October 2013 | 877 | CM,  F: 47% M:53.0%,  median: 30 and 34 | Convenience | Online survey | Unknown |  |
| Koon-Magnin, 2015 | US | two days in August of 2013 | Study 1: 188  Study 2: 242 | CM,  F:65% M:35%,  Age 19-20: 15.4%, Age 30-39: 17.2%, Age 40-49: 16.9%, Age 50-59: 19.4%, Age 60 +: 30.9% | Convenience  RDD | Paper and phone surveys | 64% |  |
| Levenson et al. 2007 | US | During August of 2005 | 193 | CM,  F: 57% M:43%,  mean:37 | NR | Questionnaire | NR |  |
| Lieb & Nunlist, 2008 | US | September and October 2007 | 643 | CM,  NR,  NR | RDD | Phone survey | NR |  |
| Manchak & Fisher, 2019 | US | NR | 253 | students,  F:58% M:42%,  26 (SD = 9) | NR | Online survey with vignettes | 19.8% |  |
| Mancini et al. 2010 | US | In spring 2006 | 1,308 | CM,  F:59.7% M:40.3%,  45-54: 24.4%, 55-64: 18.4%, 35-44: 18.0% … | Two-stage modified Mitofsky-Waksberg RDD | Telephone survey | 48.6% |  |
| Mancini, 2014 | US | April 29 to May 1 2015 | 1,006 | CM,  mean:0.49 (0.50) (1=M),  50.43 (16.34) | NR | National public opinion telephone poll | NR |  |
| Mears et al. 2008 | US | November 9, 2006, through January 6, 2007 | 425 | CM,  F: 53% M:47%,  NR | RDD | Telephone-based survey | 46.8% |  |
| Phillips, 1998 | US | NR | 400 | CM,  NR,  NR | RDD | Telephone survey | NR |  |
| Redlich, 2001 | US | NR | 269  (CM:109 Students:82) | CM, students,  NR,  CM: 46, Students: 28 | CM: random mailings and random people interested in the study | Paper and pencil questionnaires | CM: 33%,  Students:27% |  |
| Rosselli & Jeglic, 2017 | US | Four-week period in June and July 1997 | 559 | Students (psychology),  F:75% M:25%,  M=20.40 years, SD=3.55 | Convenience | Questionnaires | NR |  |
| Sample et al. 2011 | US | February 2008 and August 2008 | 1,811 | CM,  F:50.5% M:49.5%,  25 to 44: 36.6%,  45 to 64: 33.7% | Random | Survey | 38% |  |
| Schiavone & Jeglic, 2009 | US | NR | 115 | CM,  F: 85.2% M:14.8%,  25-64: 78.9% Younger than 25: 20.2% | Nonrandom self-selected sample | Online survey | NR |  |
| Shackley et al. 2014 | Australia | NR | 552 | CM,  F:73.6% M:26.4%,  29.67 years (SD 11.85) | NR | Online survey | NR |  |
| Socia & Harris, 2016 | US | NR | 1,000  (915 after dropping missing) | CM,  F:54% M:  48.21 (16.00) | Propensity score matching | Online survey | NR |  |
| Spoo et al. 2018 | US | NR | 1,173 | Students,  F: 74% M:  *M* = 20.61, SD = 6 | NR | Online survey | NR |  |
| Taylor, 2017 | Australia | June 2013 and remained open till the end of October 2013 | 162 | CM,  F:59.2% M:35.5%,  Median: 40 | NR | Online survey | NR |  |
| Zevitz & Farkas, 2000 | US | January 1998 through mid-September 1998 | 704 | CM  NR  NR | Convenience | Paper and pencil questionnaire,  Meetings observation | 88% |  |
| Zgoba & Cowan, 2020 | UK | Spring 2015 (over 3 days) | 140 | CM,  F:64.3% M:35%,  31.67 (13.64) | Random walking individuals at three different locations (Driver and Vehicle Licensing Agency, university, train station) | Survey | NR |  |

*Note:* CM: Community members; F: Female; M: Male; NR: Not reported; RDD: Random digit dialing

Appendix B.

Table 2.

*Q-SSP quality assessment score for included studies*

| QSSP | Anderson & Sample (2008) | Anderson et al. (2009) | Beck et al. (2004) | Berryessa & Lively (2019) | Branon et al (2007) | Burchfield (2012) | Button et al (2013) | Brown et al (2008) | Budd & Mancini (2016) | Cain et al (2017) | Chui et al. (2015) | Comartin et al. (2009) | Craun & Theriot (2009) | Craun (2010) | Center for Sex Offender Management (2010) | Garland et al. (2018) |
| --- | --- | --- | --- | --- | --- | --- | --- | --- | --- | --- | --- | --- | --- | --- | --- | --- |
| 1.Was the problem or phenomenon under investigation defined, described, and justified? | yes | yes | yes | yes | Yes | yes | yes | yes | yes | yes | yes | yes | yes | yes | yes | yes |
| 2.Was the population under investigation defined, described, and justified? | yes | yes | yes | not stated clearly | yes | yes | not stated clearly | yes | yes | no | yes | no | yes | yes | no | yes |
| 3.Were specific research questions or hypotheses stated? | yes | yes | yes | yes | yes | yes | not stated clearly | yes | yes | yes | yes | no | yes | yes | no | yes |
| 4.Were operational definitions of all study variables provided? | yes | yes | yes | yes | yes | yes | yes | no | yes | yes | yes | yes | yes | yes | no | yes |
| 5.Were participant inclusion criteria stated? | yes | yes | yes | not stated clearly | yes | yes | yes | yes | yes | no | yes | no | no | yes | no | yes |
| 6.Was the participant recruitment strategy described? | yes | yes | yes | yes | yes | yes | yes | yes | no | yes | no | yes | yes | yes | no | yes |
| 7.Was a justification/rationale for the sample size provided? | no | no | yes | no | no | no | no | no | no | no | no | no | no | no | no | no |
| 8.Was the attrition rate provided? | yes | yes | yes | not stated clearly | not stated clearly | yes | no | not stated clearly | no | yes | not stated clearly | not stated clearly | not stated clearly | not stated clearly | no | no |
| 9.Was a method of treating attrition provided? | no | no | yes | no | no | no | not stated clearly | no | no | no | no | no | no | no | no | no |
| 10.Were the data analysis techniques justified? | not stated clearly | not stated clearly | not stated clearly | yes | no | no | not stated clearly | no | not stated clearly | not stated clearly | not stated clearly | not stated clearly | yes | yes | no | not stated clearly |
| 11.Were the measures provided in the report (or in a supplement) in full? | yes | yes | yes | yes | not stated clearly | yes | yes | no | yes | yes | no | no | yes | yes | no | no |
| 12.Was evidence provided for the validity of all the measures (or instrument) used? | no | no | yes | no | no | yes | no | no | no | no | yes | no | yes | yes | no | no |
| 13.Was information provided about the person(s) who collected the data (e.g., training, expertise, other demographic characteristics)? | no | no | no | no | no | no | no | no | no | no | yes | no | no | no | no | yes |
| 14.Was information provided about the context (e.g., place) of data collection? | yes | yes | yes | yes | not stated clearly | yes | no | not stated clearly | no | yes | yes | no | yes | yes | no | yes |
| 15.Was information provided about the duration (or start and end date) of data collection? | yes | yes | not stated clearly | yes | no | no | no | yes | yes | yes | no | no | no | no | yes | no |
| 16.Was the study sample described in terms of key demographic characteristics? | yes | yes | not stated clearly | no | not stated clearly | not stated clearly | yes | no | yes | yes | no | yes | no | yes | no | no |
| 17.Was discussion of findings confined to the population from which the sample was drawn? | not stated clearly | not stated clearly | not stated clearly | no | not stated clearly | no | not stated clearly | no | no | yes | yes | no | not stated clearly | not stated clearly | no | no |
| 18.Were participants asked to provide (informed) consent or assent? | not stated clearly | not stated clearly | not stated clearly | yes | yes | not stated clearly | not stated clearly | not stated clearly | not stated clearly | not stated clearly | not stated clearly | not stated clearly | not stated clearly | not stated clearly | not stated clearly | yes |
| 19.Were participants debriefed at the end of data collection? | not stated clearly | not stated clearly | not stated clearly | not stated clearly | no | no | not stated clearly | no | not stated clearly | not stated clearly | not stated clearly | no | not stated clearly | not stated clearly | not stated clearly | no |
| 20.Were funding sources or conflicts of interest disclosed? | no | no | no | no | no | no | yes | no | no | yes | yes | no | no | no | yes | yes |
| Score | 55% | 55% | 60% | 45% | 35% | 50% | 35% | 30% | 40% | 55% | 50% | 20% | 45% | 55% | 15% | 50% |

Table 2.

*Q-SSP quality assessment score for included studies (continuation)*

| QSSP | Harris & Cudmore (2018) | Harris & Socia (2016) | Jung et al. (2018) | Kernsmith et al. (2009) | Kernsmith et al. (2016) | King (2019) | Klein & Tolson (2019) | Levenson et al (2007) | Lieb & Nunlist (2008) | Mancini et al. (2010) | Mancini (2014) | Mears et al. (2008) | Phillips (1998) | Rosselli & Jeglic (2017) | Redlich (2001) | Sample et al. (2011) |
| --- | --- | --- | --- | --- | --- | --- | --- | --- | --- | --- | --- | --- | --- | --- | --- | --- |
| 1.Was the problem or phenomenon under investigation defined, described, and justified? | yes | yes | yes | yes | yes | yes | yes | not stated clearly | yes | yes | yes | yes | yes | yes | yes | yes |
| 2.Was the population under investigation defined, described, and justified? | yes | no | yes | no | no | yes | no | not stated clearly | yes | yes | not stated clearly | yes | yes | not stated clearly | yes | not stated clearly |
| 3.Were specific research questions or hypotheses stated? | yes | yes | yes | yes | yes | yes | yes | yes | no | no | yes | yes | no | yes | no | no |
| 4.Were operational definitions of all study variables provided? | yes | yes | yes | yes | yes | yes | yes | yes | no | yes | yes | yes | no | yes | yes | yes |
| 5.Were participant inclusion criteria stated? | yes | yes | yes | yes | yes | yes | yes | no | yes | yes | no | yes | yes | yes | yes | yes |
| 6.Was the participant recruitment strategy described? | yes | yes | yes | yes | yes | yes | yes | yes | yes | no | no | yes | no | yes | yes | yes |
| 7.Was a justification/rationale for the sample size provided? | no | no | no | no | no | no | no | no | no | not stated clearly | no | yes | no | no | no | no |
| 8.Was the attrition rate provided? | no | no | no | not stated clearly | no | not stated clearly | no | no | no | yes | no | not stated clearly | no | no | no | not stated clearly |
| 9.Was a method of treating attrition provided? | no | no | no | no | no | no | no | no | no | no | no | no | no | no | no | no |
| 10.Were the data analysis techniques justified? | yes | not stated clearly | yes | yes | not stated clearly | no | yes | no | no | not stated clearly | not stated clearly | yes | no | no | no | no |
| 11.Were the measures provided in the report (or in a supplement) in full? | no | yes | no | yes | no | no | yes | yes | no | yes | yes | yes | no | yes | no | yes |
| 12.Was evidence provided for the validity of all the measures (or instrument) used? | no | no | no | yes | no | no | yes | no | no | no | no | no | no | no | no | no |
| 13.Was information provided about the person(s) who collected the data (e.g., training, expertise, other demographic characteristics)? | no | no | no | no | no | no | no | yes | no | no | no | yes | no | no | yes | no |
| 14.Was information provided about the context (e.g., place) of data collection? | yes | yes | yes | yes | yes | yes | yes | yes | yes | yes | yes | yes | yes | no | yes | yes |
| 15.Was information provided about the duration (or start and end date) of data collection? | yes | yes | no | no | no | no | yes | yes | yes | yes | yes | yes | yes | no | no | not stated clearly |
| 16.Was the study sample described in terms of key demographic characteristics? | yes | yes | not stated clearly | not stated clearly | yes | yes | no | no | no | yes | yes | yes | no | no | no | yes |
| 17.Was discussion of findings confined to the population from which the sample was drawn? | yes | not stated clearly | yes | not stated clearly | not stated clearly | not stated clearly | not stated clearly | yes | yes | not stated clearly | not stated clearly | yes | yes | no | yes | yes |
| 18.Were participants asked to provide (informed) consent or assent? | no | no | yes | no | yes | yes | yes | yes | no | no | no | no | no | yes | no | no |
| 19.Were participants debriefed at the end of data collection? | no | no | yes | no | no | not stated clearly | no | no | no | no | no | no | no | yes | no | no |
| 20.Were funding sources or conflicts of interest disclosed? | yes | yes | no | no | no | yes | yes | no | no | not stated clearly | no | no | no | yes | not stated clearly | yes |
| Score | 60% | 50% | 55% | 45% | 40% | 50% | 60% | 45% | 35% | 45% | 35% | 70% | 30% | 45% | 40% | 45% |

Table 2.

*Q-SSP quality assessment score for included studies (continuation)*

| QSSP | Schiavone & Jeglic (2009) | Shackley et al. (2014) | Socia & Harris (2016) | Spoo et al (2018) | Taylor (2017) | Zgoba & Cowan (2020) | Zevitz & Farkas (2000) |
| --- | --- | --- | --- | --- | --- | --- | --- |
| 1.Was the problem or phenomenon under investigation defined, described, and justified? | yes | yes | yes | yes | yes | yes | yes |
| 2.Was the population under investigation defined, described, and justified? | yes | yes | not stated clearly | yes | yes | yes | yes |
| 3.Were specific research questions or hypotheses stated? | no | yes | yes | yes | no | yes | yes |
| 4.Were operational definitions of all study variables provided? | yes | yes | yes | yes | yes | yes | no |
| 5.Were participant inclusion criteria stated? | no | yes | yes | yes | yes | yes | yes |
| 6.Was the participant recruitment strategy described? | yes | yes | yes | yes | yes | yes | yes |
| 7.Was a justification/rationale for the sample size provided? | no | no | no | no | no | no | no |
| 8.Was the attrition rate provided? | no | no | no | no | no | no | not stated clearly |
| 9.Was a method of treating attrition provided? | no | no | no | no | no | no | no |
| 10.Were the data analysis techniques justified? | no | yes | yes | yes | no | no | no |
| 11.Were the measures provided in the report (or in a supplement) in full? | yes | yes | yes | no | yes | no | yes |
| 12.Was evidence provided for the validity of all the measures (or instrument) used? | no | yes | no | no | no | no | no |
| 13.Was information provided about the person(s) who collected the data (e.g., training, expertise, other demographic characteristics)? | no | no | no | no | no | no | no |
| 14.Was information provided about the context (e.g., place) of data collection? | not stated clearly | not stated clearly | yes | yes | yes | yes | yes |
| 15.Was information provided about the duration (or start and end date) of data collection? | no | no | yes | no | yes | not stated clearly | yes |
| 16.Was the study sample described in terms of key demographic characteristics? | yes | not stated clearly | yes | not stated clearly | no | yes | no |
| 17.Was discussion of findings confined to the population from which the sample was drawn? | yes | yes | not stated clearly | not stated clearly | yes | yes | not stated clearly |
| 18.Were participants asked to provide (informed) consent or assent? | no | yes | no | yes | no | yes | no |
| 19.Were participants debriefed at the end of data collection? | no | not stated clearly | no | yes | no | yes | no |
| 20.Were funding sources or conflicts of interest disclosed? | no | no | no | yes | yes | yes | no |
| Score | 35% | 55% | 50% | 55% | 50% | 60% | 40% |

Appendix C.

Table 3.

*Public Perceptions and Actions about the* *Community Management Policies*

| Author(s), year | Support of policies  (*k*=24) | Effectiveness/  Usefulness of policies  (*k*=18) | Feeling safe  (personally, family, community)  (*k*=12) | Knowledge of negative/collateral consequences for ICSO  (*k*=11) | Access of registry  (*k*=8) | Taken preventive action  (*k*=8) | Familiarity/  awareness of policies  (*k*=9) | Fear of ICSO/ having ICSO in the neighborhood (*k*=8) | Other |
| --- | --- | --- | --- | --- | --- | --- | --- | --- | --- |
| Anderson & Sample, 2008^*^ |  |  |  |  |  |  |  |  | Times accessed the registry |
| Anderson et al. 2009 |  |  |  |  |  |  |  |  |  |
| Beck et al., 2004^*^ |  |  |  |  |  |  |  |  |  |
| Berryessa & Lively, 2019 |  |  |  |  |  |  |  |  | Social distancing |
| Beshears, 2017 |  |  |  |  |  |  |  |  | Knowledge of the offender/offense, Perceptions on notification process, Effects on lifestyle of residents with registered ICSO |
| Brannon et al. 2007^*^ |  |  |  |  |  |  |  |  | Fairness of policies, Notification method most effective |
| Brown et al. 2008 |  |  |  |  |  |  |  |  | Information source about ICSO, Fear of becoming a victim of sexual offense, Information about past sexual offenses if living near a ICSO |
| Burchfield, 2012^*^ |  |  |  |  |  |  |  |  | Awareness of ICSO living in the neighborhood~~,~~ Likelihood of neighbor reporting ICSO, Trust on registered offenders, Negative opinions of registered offenders, Residential awareness and Reporting local ICSO, Informal social control, Social ties, Neighborhood attachment, Neighboring behaviors, Neighborhood disadvantage, Neighborhood immigrant concentration, Neighborhood residential instability |
| Budd & Mancini, 2016 |  |  |  |  |  |  |  |  | Misconceptions, Legislators should rely on media and the public |
| Cain et al. 2017^*^ |  |  |  |  |  |  |  |  | Females commit sex crimes, and are less serious than men sex crimes, Type of preventive actions |
| Campbell & Newheiser, 2019^*^ |  |  |  |  |  |  |  |  | Reasons to held the attitudes toward these laws even participants learnt they were ineffective (Irredeemability, Mixed–Pro, Mixed-Pro/con), Persuasion to change their attitudes |
| CSOM, 2010^*^ |  |  |  |  |  |  |  |  | Factor that is (and should be) most influential for guiding lawmaker decisions on ICSO management policies, Attitudes about management policies in relation to research findings, More information needed |
| Chui et al., 2015^*^ |  |  |  |  |  |  |  |  | Longer prison sentences, Rehabilitation views, Stereotypical views |
| Comartin et al., 2009^*^ |  |  |  |  |  |  |  |  |  |
| Craun & Theriot, 2009 |  |  |  |  |  |  |  |  |  |
| Craun, 2010^*^ |  |  |  |  |  |  |  |  | Awareness of neighborhood registered ICSO, Fear of crime, Knowledge of community crime, SES, Ethnic heterogeneity in neighborhood, Residential stability, Family structure, Urbanization |
| Garland et al. 2018 |  |  |  |  |  |  |  |  | Notification method most effective, Information in the registry |
| Harris & Cudmore, 2018^*^ |  |  |  |  |  |  |  |  | Reasons for use/non use the registry |
| Harris & Socia, 2016^*^ |  |  |  |  |  |  |  |  | Amenability to rehabilitation, Juvenile risk and registration |
| Jung et al., 2018^*^ |  |  |  |  |  |  |  |  |  |
| Kernsmith et al., 2009^*^ |  |  |  |  |  |  |  |  |  |
| Kernsmith et al., 2016^*^ |  |  |  |  |  |  |  |  |  |
| King, 2019^*^ |  |  |  |  |  |  |  |  |  |
| Klein & Cooper, 2019^*^ |  |  |  |  |  |  |  |  | Moral panic (concern, hostility, consensus, volatility, and disproportionality) |
| Koon-Magnin, 2015^*^ |  |  |  |  |  |  |  |  | Notification method most effective, Anger, Research influence on their policies’ views/support, punitive attitudes |
| Levenson et al., 2007^*^ |  |  |  |  |  |  |  |  | Notification method most effective, Research influence on their policies’ views/support |
| Lieb & Nunlist, 2008^*^ |  |  |  |  |  |  |  |  | How respondents learned of community notification law, Awareness of ICSO living in the community, Juvenile notification, Angry, |
| Manchak & Fisher, 2019 |  |  |  |  |  |  |  |  | Perceptions of riskiness of offender, Social distancing |
| Mancini et al., 2010^*^ |  |  |  |  |  |  |  |  |  |
| Mancini, 2014^*^ |  |  |  |  |  |  |  |  | Rehabilitation views |
| Mears et al., 2008^*^ |  |  |  |  |  |  |  |  | Support for making sex crimes a policy priority, Most appropriate punishment for a ICSO against children |
| Phillips, 1998^*^ |  |  |  |  |  |  |  |  | Awareness of ICSO in neighborhood, Learned more about ICSO and their modus operandi, Police should notify citizens, Purpose of community notification |
| Redlich, 2001^*^ |  |  |  |  |  |  |  |  | The degree to which community notification laws directly violate offenders’  Rehabilitation views, Angry if drug felon/child molester/murderer living in the neighborhood |
| Rosselli & Jeglic, 2017^*^ |  |  |  |  |  |  |  |  | Fairness of policies, The information listed on the Internet registry  helps the public to protect themselves, ICSO manage their risk because neighbors are watching them, Rehabilitation views |
| Sample et al., 2011^*^ |  |  |  |  |  |  |  |  | Reasons for (not)accessing the registry |
| Schiavone & Jeglic, 2009^*^ |  |  |  |  |  |  |  |  | Source of information of registration/ notification laws, Information in the registry, Fairness of policies |
| Shackley et al., 2014 |  |  |  |  |  |  |  |  |  |
| Socia & Harris, 2016^*^ |  |  |  |  |  |  |  |  | Risk estimate, Increase funding for the registry, Sex crimes estimation, Believe university research, Research would not change laws views |
| Spoo et al., 2018 |  |  |  |  |  |  |  |  | Rehabilitation views |
| Taylor, 2017^*^ |  |  |  |  |  |  |  |  | Deserving, Information in the registry, Website usability (easy to understand, easy to find, easy to use) and utility (ICSO rights, community rights, protection, help police, availability in all states, provides safety for children) |
| Zevitz & Farkas, 2000 |  |  |  |  |  |  |  |  | Method attendees were alerted to the notification meetings, Purpose of the meeting, outcome expectations about the meeting, Information received, Level of concern |
| Zgoba & Cowan, 2020^*^ |  |  |  |  |  |  |  |  | How respondents found out about the notification scheme, Public perceptions of disclosure law (No notification should be made to the public, What ICSO should be subject to the law), Perceived effectiveness (ICSO feel shame so reduce reoffending, these laws make ICSO more honest with people they know, Rehabilitation views, Fairness of policies,  Research would not change laws views |

*Note:* CSOM = Center for Sex Offender Management; ICSO = Individuals convicted of sex offenses; *k* = number of studies.

^*^Included in the meta-analysis. Due to Anderson & Sample (2008) and Anderson et al. (2009) used the same sample, only the former study was included

Appendix D.

Table 4.

*Agreement Proportions on Knowledge and Misconceptions Items about ICSO and the Community Management Policies*

| Study | Instrument | Recidivism rate | Sexual offense rates are on the rise | Levels of risk of ICSO | ICSO stranger or related to the victim | Treatment programs are a waste of money/time | Information source about ICSO/policies | Support law even no evidence | Other | Total score |
| --- | --- | --- | --- | --- | --- | --- | --- | --- | --- | --- |
| Brown et al. 2008 | Questionnaire  (5 sections) | 0-20% reoffend:  32%  26-50%:  36.5%  51-75%:26%  76-100%:  5.5% |  | Media were: accurate (32%), exaggerated (38%) underestimated (30%) the risk |  |  | Media:  (% NR) |  | Media were: accurate (39% agree), exaggerated (45.5%) underestimated (15.5%) the risk of becoming a victim |  |
| Budd & Mancini, 2016 | KQ (CSOM, 2000): 4-item | Recidivates with a sex crime: 57%  Recidivates with a more serious, more violent crime: 35% |  |  | Stranger: 8% |  | Media: 74%  Internet: 8% |  | Effectiveness of sex offender treatment in reducing recidivism (very/somewhat effective): 61%  Legislators should rely on media: 5%  Legislators should rely on the public: 19% |  |
| Campbell & Newheise, 2019 | Questionnaire  (7-items) | On average the range was 50-75% |  |  | On average the range to be stranger to victim was 25-50% |  |  | Law support even after exposure to counterevidence |  |  |
| CSOM, 2010 | Survey | <25%: 3%  25-50%:19%  50-75%: 39%  >75%: 33% |  |  | Stranger: 8% |  | (News)Media: 74%  Internet: 8% | 56% support residence restriction even unintended consequences.  43% support prison even research demonstrate alternatives to prison are more effective | What the public wants is the most important factor for guiding lawmakers’ decisions about laws: 38%  Research on effective strategies should be the most important factor for guiding lawmakers’ decisions about laws: 58% |  |
| Craun & Theriot, 2009 | Victimization Scale (Coulton, Korbin, and Su, 1996): 2 items |  |  |  | 30% were more concern about stranger perpetrator compared to someone they knew |  |  |  |  |  |
| Lieb & Nunlist, 2008 |  |  |  |  |  |  | TV/Radio: 63%  Newspapers: 51% |  | I learned more about ICSO and their modus operandi because of community notification: 68% |  |
| Jung et al. 2018 | Knowledge of the registry (Jung et al. 2018): 11 brief scenarios |  |  | Americans (44%) were more knowledgeable than Canadians (43%) in identifying ICSO who would be  placed on the registry |  |  |  |  |  |  |
| Kernsmith et al. 2016 | KQ(CSOM, 2000): 11-item scale |  |  |  |  |  |  |  |  | M = 2.41, SD = 0.29^1^ |
| King, 2019 | PASO (King, 2019): 4-items measured misconception (out of 12items) | 63% agreed that almost ICSO will reoffend | 70% |  |  | 25% |  |  | Teaching children about “stranger danger” is one of  the best ways to protect them from sex offenders: 82% | M= 44.56, SD = 8.29^2^ |
| Koon-Magnin, 2015 | Same as Levenson, et al. (2007, see below) |  | Paper/phone survey: 58% / 67% |  |  | Paper survey:  46% |  | Paper/phone survey:  58% /72% | Substance use play a moderate/major role in sex offending: 48%  Abuse only occurs in low socio-economic classes: 11%  ICSO reoffend at much higher rates than other offenders: 48% |  |
| Levenson et al. 2007 | KQ: 11-item scale  Five statements about ICSO treatment, sex crime rates, and ICSO characteristics | Mean rate estimations of ICSO recidivism: 74% | 77% | All ICSO should be subject to notification: 76%, | 49% by strangers  58% of boys abused by known person  63% of girls abused by known person | 50% |  | 73% (completely true: 49%) | Substance use play a moderate/major role in sex offending: 65%  Abuse only occurs in low socio-economic classes: 7%  ICSO reoffend at much higher rates than other offenders: 68%^3^ | KQ: 59% (over 100%)  Statements: 53% |
| Manchak & Fisher, 2019 | KQ (CSOM, 2008): 6-item |  |  |  |  |  |  |  |  | Correct 4 or + questions: 87%^4^ |
| Redlich, 2001 | Child  Abuse Knowledge Questionnaire.  Child Abuse Prevention Questionnaire (CAPQ, Redlich, 2000). |  |  |  |  | Likelihood of child molesters  being rehabilitated: M=0.82 (CM), M=0.84(students)  Likelihood of people who sexually  assault adults being rehabilitated: M=0.93 (CM), M=1.01(students)^5^ |  |  | Regular measures violate rights: M=0.90(CM), M=1.48(students)  Drastic measures violate rights: M=2.10(CM), M=2.59(students)^6^ |  |
| Rosselli & Jeglic 2017 | KQ (CSOM, 2002): 26-item |  |  | 81% disagree on “small number of ICSO are dangerous” |  | 20% |  |  | 41% agree that ICSO should lose their civils rights | KQ(*M*=16.64 *SD*=2.43)^7^, |
| Schiavone & Jeglic 2009 | MLS (Levenson & Cotter 2005)  Attitude/Perception Toward Sex Offenders and Sex Offender Policies Survey (Schiavone & Jeglic, 2009) | 88% agree on “if they want to reoffend, they would able to do despite the residence restrictions” |  | High (89%), moderate (82%) and low (51%) risk offenders should be subjected to the notification and registration. 20% felt ICSO posing no risk should also be subjected to this law. |  |  | TV: 44%, Internet: 38% |  | 80% felt registration and notification were constitutional.  75% believed that this law did not violate ICSO right to privacy.  37% felt that ICSO have no rights |  |
| Shackley et al. 2014 | 5-items to assess community attitudes towards registration, community notification and residency restriction policies |  |  | All SO should be subject to registration (% NR) |  |  |  |  |  |  |
| Socia & Harris, 2016 | Risk Perception: 15-item + 3 questions about beliefs and actions | 50% to >90% at risk of sexual recidivism: 83%  50% to >90% at risk of non-sexual recidivism: 53% | 74% | 50% believed that 50% or more registered ICSO were at high risk of abducting children, having future child victims. | 50% stranger: 32%,  25% stranger: 25%,  75% stranger: 13% |  |  | M=0.58(SD=0.49)^8^ | Believe university research: M=1.75(SD=0.78)^9^  70% believed that 50% or more registered ICSO were pedophiles or at high risk of having future teen/adults victims.  75% believed that 50% or more were sexual predators | Risk perception of registered ICSO: M=3.07(SD=0.97)^10^ |
| Spoo et al., 2018 | KQ (CSOM, 2000): 26-item  ATTSO: 15 item (only Treatment Ineffectiveness subscale included) |  |  |  |  | Victims M=8.07 (SD=2.43), Non-victims (M= 8.00 (SD=2.2) |  |  |  | KQ:  Victims (M = 28.4, SD = 2.05)  Non-victims (M = 27.98, SD = 2.16), |
| Taylor, 2017 | Section 3 consisted in 16 item-questionnaire on attitudes and perceptions of the website |  |  | Many respondents believed that all ICSO should be placed in the registry (% NR) |  |  |  |  | Convicted child sexual  offenders have a right to anonymity and privacy of  where they live: 25% (strongly)agreed |  |
| Zgoba & Cowan, 2020 | 20 item questionnaire (Levenson et al., 2007) | 57% of ICSO would reoffend  62% believe ICSO reoffend at higher rates than non ICSO | 36% | All ICSO should be in the registry: 46% | 36% by stranger | 81.4%  felt that mandatory treatment in prison  would be helpful in reducing child sexual abuse | TV news: 37%  Work/school: 28% | 39% |  |  |

*Note.* ATTSO= Attitudes towards Sex Offenders Treatment (Rogers et al., 2011; Wnuk et al., 2006); CATSO= (Conley, Hill, Church, Stoeckel, & Allen, 2011; Rogers et al., 2011); CSOM = Center for Sex Offender Management; CNS = Community Notification Survey; CSOM = Center for Sex Offender Management; ICSO = Individuals convicted for sex offenses; KQ = Sex Offender Knowledge Quiz; MLS=Megan’s Law Survey-Community Based Revision (Levenson et al., 2007); PASO = Perceptions about Sexual Offenses Scale.

^1^ Items were measured on a four-point Likert-type scale (1 = definitely true and 4 = definitely untrue). Higher scores indicated higher levels of misinformation (Kernsmith et al., 2016).

^2^ Scores ranged from 21 to 60, midpoint of 36 including the 12 items (4 measured misconceptions and 8 punitiveness) (King, 2019).

^3^Other perceptions were about: percentage of different types of ICSO will reoffend (rapist: 74%, child molesters: 76%), percentage of ICSO abused in their childhoods (67%), percentage of ICSO were severely mentally ill (50%), percentage of children find sex with an adult a positive experience (12%), percentage of sex offenders are male (77%), percentage of sex offenders come to the attention of authorities (46%) (Table 3, Levenson et al., 2007).

^4^Correct responses scored 1 point (or 0 otherwise), so higher scores indicted more accurate knowledge. This study also measured perception of riskiness but data was not reported (Manchak & Fisher, 2019).

^5,6^ These questions were scored as 0 = not at all likely/ does not directly violate rights, 3 = very likely/ directly violates rights. Regular measures were notification and registration and drastic measures involved harming known child molester, to wear a T-shirt stating “I am a convicted child molester”, etc. (Redlich, 2010).

^7^Correct responses scored 1 point, so higher scores indicted more accurate knowledge. Additionally, 3 variables (rehabilitation is a waste of time, only a few ICSO are dangerous, and ICSO should lose their civil rights) are from the CATSO scale.

^8,9,10^ Higher values indicate high risk estimate (range from 1 to 5, 0 to 1, and 0 to 3; respectively) (Socia & Harris, 2016).

Appendix E.

Table 5.

*Factors Associated to the Public Attitudes and Perceptions of Policies*

| Author(s), year | DV variables | Factors/predictors examined | Factors associated |
| --- | --- | --- | --- |
| Correlates | | | |
| Anderson & Sample, 2008 | 1) Know of registry  2) Access registry  3) Times accessed  4) Will keep family safe  5) You feel safer  6) Preventive action taken | 1. Sex 2. Age 3. Marital status 4. Parental status 5. Education 6. Income 7. Race 8. Living area | 1) Female(+), age, marital status, having children (+), education, $20,000 or more, Caucasian  2) Female(+), age, marital status and having children, education, $20,000 or more, city  3) Marital status  4) None  5) Age, having children  6) Female(+), having children |
| Comartin et al., 2009 | 1) Support for offender policies (i.e., residency/work restrictions, community notification, controlling movement)  2) Severe policies (i.e., life in prison, castration) | - Fear of ICSO  - Income  - Education  - Parental status  - Race  - Home ownership  - Duration at the address  - Age  - Knowing a victim of a sex crime  - Previous criminal conviction | 1) Fear of ICSO, income(-), education(-), having children()  2) Fear of ICSO, income(-), education(-), having children() |
| Harris & Cudmore, 2018 | 1) Any registry use  2) Used registry 3+ times  3) Used registry > 5 times | - Sex  - Marital status  - Parental status  - Education  - Race  - Religious importance  - Age  - Political ideology  - Income  - Sexuality  - Living region | 1) Female, Caucasian, married, having children, living region, political ideology,  2) age, married and having children  3) married, having children, political ideology, religion |
| Jung et al., 2018 | 1) Attitudes/BJW  2) Attitudes/CATSO_social isolation  3)Attitudes/CATSO_capacity to change  4)Attitudes/CATSO_severity  5)Attitudes/CATSO_deviance  6) Political attitudes/SPA_total  7) Political attitudes/SPA_political compassion  8) Political attitudes/SPA_wealth distribution  9) Political attitudes/SPA_individual rights | Perspectives of the registry:  -View 1: register should  be available to the  public  - View 2: register should be  available to law  enforcement  - View 3: register  protect the  general public  - View 4: register help  offenders live in the  community  - View 5: register prevent  commission of sex  offenses  - View 6: register prevent  commission of non-sex  offenses  - View 7: ICSO  should have residential  restrictions  - Knowledge on registry | 1) Views 3, 5, 6  2) Views 2, 4, 5, 6, knowledge  3) Views 1, 3, 4, 7, knowledge  4) Views, 2, 4, 5, 6  5) Views 2, 4, 5, 6, knowledge  6) Views 2, 4, 7, knowledge  7) Views 2, 6, knowledge  8) Views 1, 7, knowledge  9) Views 2, 4, 6 |
| Kernsmith et al., 2009 | 1) Fear level for incest  2) Fear level for statutory rape  3) Fear level for juvenile offender  4) Fear level for marital rape  5) Fear level for pedophile  6) Fear level for date rape  7) Fear level for old offense | - Requirement to Register for incest  - Requirement to Register for statutory rape  - Requirement to Register for juvenile offender  - Requirement to Register for marital rape  - Requirement to Register for pedophile  - Requirement to Register for date rape  - Requirement to Register for an old offense | 1) all except for requirement to register for statutory rape  2) all  3) all  4) all  5) all  6) all  7) all |
| Kernsmith et al., 2016 | 1) Policy support  a) Community notification  b) Severe (life in prison, castration) | - Fear  - Misinformation  - Victimization  - Parental status  - Sex  - Education | 1a) Fear(+), misinformation(+), education(-)  1b) Fear(+), misinformation(+), education(-) |
| Manchak & Fisher, 2019 | Support for ICSO policy | - Sex  - Age  - Race  - Parental status  - Political orientation  - Knowledge about ICSO  - Beliefs about effectiveness of policy: Improves public and personal safety, causes harm to the offenders  - Perceptions of riskiness  - Social distancing attitudes  - Offense characteristics: offender sex, offender criminal history, victim age | Beliefs about effectiveness of policy, perceptions of riskiness, social distancing attitudes, offender criminal history, victim age |
| Rosselli & Jeglic, 2017 | Knowledge about ICSO  Attitudes towards ICSO  Attitudes towards ICSO treatment  Attitudes toward community notification laws  Authoritarianism–conservatism–traditionalism model | NA | Decreased knowledge was significantly  related to increased negative attitudes toward  ICSO, sex offender treatment, and  community notification laws.  No significant relationship was  found between conservative beliefs and attitudes towards community notification laws. |
| Spoo et al., 2018 | Knowledge about ICSO  Attitudes towards ICSO  Attitudes towards ICSO treatment  Attitudes toward community notification laws | NA | Greater knowledge significantly correlated with positive attitudes towards ICSO, treatment, and against the community notification laws, and the residence restriction laws |
| Redlich, 2001 | Knowledge about child sexual abuse  Support for the notification and registration of ICSO | NA | Greater knowledge was associated with:   - less supportive of the offender notification law and residence restriction laws - less supportive of all criminals should be required to register and be subject to notification - less likely to view current prevention programs as effective - more likely to think that drastic sanctions violate their rights |
| Zgoba & Cowan, 2020 | Fairness of ICSO laws  Tolerance of a ICSO living in the respondent’s neighborhood  Belief that law will reduce sexual abuse  Belief that mandatory treatment will reduce sexual abuse | - Sex  - Age  - Race  - Education  - Parental status  - Sexually abused before 15 years | 1)White, above the age of 25 years or had children were more likely to perceive current  laws to be fair  2) Respondents above the age of 25  years were more likely to be less tolerant of a sex offender living in their neighborhood,  3)whereas respondents below the age of 25 years are more likely to believe the current disclosure schemes are useful in reducing child sexual offenses  4)White(+) |
| Predictors | | | |
| Anderson et al. 2009 | 1) Access registry  2) Female accessed registry  3) Male accessed registry  4) Preventive action taken  5) Female preventive action taken  6) Male preventive action taken | - Sex  - Age  - Marital status  - Parental status  - Education  - Income  - Race  - Living area  - Internet access | 1) Female, age, education, having children, living area, internet access  2) Age, education(+), living area, internet access  3) Age, having children, living area, education, internet access  4) Sex, education, having children, internet access  5) Education, having children, living area  6) Education, internet access |
| Budd & Mancini, 2016 | Support residence restrictions even it is ineffective | -Sex  -Age  -Race  -Parental status  -Marital status  -Education  -Income  -Religion  -Misconceptions: stranger danger, recidivism, rehabilitation effectiveness  - Primary sources of knowledge about ICSO | Catholic(+), parent(+), believe in stranger danger(+), treatment effectiveness in reducing recidivism(+) |
| Burchfield, 2012 | 1) Awareness of local ICSO  2) Neighbor’s reporting of local ICSO | - Familiarity with Illinois sex offender laws  - Deserving the laws  - Informal social control  - Income  -Trust  - Negative opinions  - Secrecy  - ICSO in neighborhood  - Social ties  - Neighborhood attachment  - Neighboring  - Neighborhood disadvantage  - Neighborhood ethnic heterogeneity  - Neighborhood  - Residential instability  - Parental status  - Marital status  - Education  - Length of residence in neighborhood | 1) Familiarity with laws(+), deserving the laws(+), informal social control(-), income (+)  2) Secrecy(+), informal social control(-), length of residence in neighborhood(+) |
| Button et al., 2013 | 1) General views about GPS monitoring of ICSO  2) Extremely positive views about the success potential of GPS monitoring of ICSO  3) Extremely negative views about the success potential of GPS monitoring of ICSO | - Sex  - Age  - Income  - Race  - Education  - Vulnerable populations in neighborhood  - Sufficiency of resources  - Child victim of violence  - Property victim  - Violence victim  - Social capital factor  - Collective efficacy  - Perceived incivility in neighborhood  - Rate of registered ICSO in ZIP code | 1) Female(+), presence of vulnerable populations  2) Female (+), Income(-),Caucasian(-), presence of vulnerable populations factor(+), perceived incivility in neighborhood(-)  3) Education(+), property victim(+), social capital factor(+), presence of vulnerable populations factor(-), rate of registered ICSO in ZIP code(+) |
| Cain et al., 2017 | 1) Taking preventive action if female who committed sex offenses lived in community(yes/no)  2) Taking preventive action if female who committed sex offenses lived in community (I do not know)  3) Female sex crimes are less serious than sex crimes committed by men  3a. “(Strongly)Agree”  3b. “(Strongly)Disagree”  3c. “Neither Agree nor Disagree”  3d. “Do Not Know” | - Sex  -Age  - Education  - Income  - Marital status  - Parental status  - Living area  - Race | 1) Female(+), high education(-), children (+)  2) Female(+), <50years old(-), married(-), Caucasian (+)  3a) Female(-), <50 years old (-), urban living area (-)  3b) Female(+), <50years old(+), high education(+), Caucasian(+)  3c) Married(+), Caucasian(+)  3d) Female(-), <50years old (+) |
| Campbell & Newheiser, 2019 | Supporting policies not based on evidence | -Sex  -Age  -Parental status  -Political ideology | Political ideology(+) |
| Comartin et al., 2009 | 1) All policies (i.e., residency/work restrictions, community notification, controlling movement)  2) Severe policies (i.e., life in prison, castration) | - Fear of ICSO  - Income  - Education  - Parental status  - Race  - Home ownership  - Duration at the address  - Age  - Knowing a victim of a sex crime  - Previous criminal conviction | 1) Fear of ICSO, education  2) Fear of ICSO, education |
| Craun & Theriot, 2009 | Misperception of ICSO risk | - Aware of ICSO in neighborhood  - Very familiar with Megan’s Law  - Worry of victimization  - Victim of violent crime  - Parental status  - Marital status  - Sex | Aware of ICSO in neighborhood (+)  Worry of victimization (+)  Victim of violent crime (+)  Being married (+)  Control group no significant model |
| Craun, 2010 | 1) Aware of registered ICSO in the neighborhood | - “Very familiar” with Megan’s Law  - Knowledge of community crime  - Victim of violent crime  - Marital status/married  - “Very familiar” with fictitious policy  - Number of address changes made by offender  - Number of offenders within 0.1 mile of respondent  - Concentrated disadvantage in neighborhood  - Residential stability  - Hispanic immigrants | 1) Very familiar” with Megan’s Law, Knowledge of community crime, “Very familiar” with fictitious policy, Number of address changes made by offender, Number of offenders within 0.1 mile of respondent, Hispanic immigrants |
| Harris & Cudmore, 2018 | 1) Any registry use  2) Used registry 3+ times  3) Used registry >5 times | - Sex  - Marital status  - Parental status  - Education  - Race  - Religion  - Age  - Political ideology  - Income | 1) Caucasian(+), children(+), living region, moderate ideology(-), age(30-59)(+)  2) never married(-), children(+), age(19-59)(+), religion very important (-), moderate ideology(-)  3) never married(-), religion very important(-), moderate(+)/liberal ideology(-), age(19-59)(+) |
| Kernsmith et al., 2009 | 1) Fear level for incest  2) Fear level for statutory rape  3) Fear level for juvenile offender  4) Fear level for marital rape  5) Fear level for pedophile  6) Fear level for date rape  7) Fear level for old offense | - Sex  - Race | None |
| King, 2019 | 1) Punitiveness  2) Misconceptions | - Misconceptions (stranger danger, sex offense rates up, almost all recidivate, treatment is a waste)  - Sex  - Age  - Race  - Education  - Income  - Parental status  - Direct victimization  - Indirect victimization  - Know convicted sex offender  - Survey mode | 1) Misconceptions(all)(+), female(+)  2) Education(-), survey mode(-) |
| Koon-Magnin, 2015 | 1) Support for community notification policies  2) Perceived effectiveness of community notification policies on reducing sexual reoffending | - Sex  - Parental status  - Race  - Age  - Education  - History of sexual victimization | 1) None  2) Male(+) |
| Manchak & Fisher, 2019 | Support for ICSO policy | - Social distancing  - Perceptions of risk  - Enhances public safety  - Causes harm to offenders  - Offender criminal history  - Victim age | All |
| Mancini et al., 2010 | Parental support for residence restrictions | - Have children  - Nº children: 1, 2, 3+  Control variables  - Sex  - Age  - Race  - Latino ethnicity  - Education  - Political ideology  - Income | Having 3+ children(+).  The effect is the same for mothers and parents, from different races/ethnic and age groups. |
| Mancini, 2014 | (Reduced) concern about unintended effects of the registry (i.e., harassment) | - Sex  - Age  - Race  - Education  - Political ideology  - Income  - Parental status  - Living area  - Used registry  - Rehabilitation views: not possible, less possible than other offenders | Used registry, (not possible) rehabilitation views |
| Mears et al., 2008 | Support for tougher sanctioning of child pornography accessers | - Sex  - Race  - Marital status  - Education  - Income  - Political ideology  - Concerned about crime | Males, Whites, the less well educated, the less wealthy, and those who were more concerned about crime |
| Redlich, 2001 | 1) Support Megan’s law  2) Knowledge about child abuse   1. the likelihood of child molesters could be rehabilitated 2. all criminals should register and be subject to notification laws 3. the likelihood that community members will 4. harm known child molesters, 5. the likelihood of rehabilitation for ICSO who abuse adults. | - Sex | 1) Women(+)  2a) Women(+) |
| Sample et al., 2011 | 1) Did not access the registry because had no interest  2) Did not access because received information from another source  3) Accessed for safety  4) Accessed out of curiosity/personal interest | - Sex  - Age  - Marital status  - Education  - Parental status  - Race  - Living area  - Income | 1) Men(+), any age(+), who had received some college(+), and with income >US$ 20.000 (+)  2) Women (+) and Whites (+)  3) Being female, divorced/separated and having children at home  4) women(-), married(-), having children at home(-), an annual household income of more than US$20,000 |
| Socia & Harris, 2016 | IRSO risk perception | - Sex  - Race/ethnicity  - Age  - Marital status  - Education  - Living area  - Political ideology  - Religion  - Political knowledge  - Believe university research  - Ever used the registry  - Registry is effective  - Increase funding for registry  - Sex crimes more common today  - Research wouldn’t change SORN views | Female(+), Hispanic(+), more conservative(+), believing the registry was effective(+), supporting increased funding for the registry(+), and believing sex crimes increased over the last 20 years(+), believing that research would not change an individual’s views about SORN(+)  Being college educated(-), with more general political knowledge(-), having ever used the registry(-) |
| Spoo et al., 2018 | 1) Community notification laws  2) Residency restriction laws  3) Attitudes/CATSO_total  4) Attitudes/CATSO_ severity  5) Attitudes/CATSO_deviancy  6) Attitudes/CATSO_social isolation  7) Attitudes/CATSO_capacity to change  8) Attitudes/ ATTSO_total  9) Attitudes/ ATTSO_incapacitation  10) Attitudes/ ATTSO_treatment ineffectiveness  11) Attitudes/ ATTSO_mandated treatment | - Knowledge of ICSO  - History sexual abuse | 1) Knowledge (-)  2) Knowledge (-)  3) Knowledge (-) and history of sexual abuse (+)  4) Knowledge (-) and history of sexual abuse (+)  5) Knowledge (-) and history of sexual abuse (+)  6) Knowledge (-)  7) Knowledge (-)  8) Knowledge (-)  9) Knowledge (-)  10) Knowledge (-)  11) Knowledge (-) |
| Moderators | | | |
| Harris & Socia, 2016 | 1) Internet registration  2) Residence restrictions  3) Treatment/impulse control  4) Social network ban  5) Internet ban  6) Juvenile registration  7) Juvenile risk | -Sex  - Age  - Education | 4) Female (+) |
| Rosselli & Jeglic, 2017 | 1) Knowledge about ICSO  2) Attitudes towards ICSO  3) Attitudes towards ICSO rehabilitation  4) Attitudes toward ICSO legislation | Conservative beliefs | Conservative beliefs moderated the relationship  between knowledge and attitudes.  Conservative beliefs did not moderate the relationship between knowledge and  attitudes toward treatment of ICSO.  Moderation analyses not run since knowledge and conservative beliefs did not predict attitudes  toward community notification laws. |
| Mediators | | | |
| Kernsmith et al., 2016 | 1) Policy support  a) Community notification  b) Severe (life in prison, castration) | - Fear  - Misinformation  - Victimization  - Parental status  - Sex  - Education | Those who showed greater misinformation and fear were also more likely to support community  notification and severe sanctions. No direct relationship was found between victimization and support for community notification and severe sanctions but victimization (especially female and older) was significantly related to fear. Parents showed higher fear, and misinformation was higher among those with lower education |
| Manchak & Fisher, 2019 | Support for policy | Victim age (IV)  Criminal history (IV)  Perception of riskiness (mediator)  Social distancing (mediator) | Social distancing and perceptions of risk partially mediated the effects of offender criminal history and victim age on support for sex offender policy |

*Note*: ATTSO =Attitudes Toward Sex Offender Treatment measure; BJW = Belief in a Just World measure; CATSO = Community Attitudes Towards Sex Offenders measure; DV = dependent variable; GPS = global positioning satellite; ICSO = individuals convicted of sexual offenses; IRSO = individuals registered for sex offenses; IV = independent variable; SORN = sex offender registration and notification; SPA = Survey of Political Attitudes measure; (+), (-) Positive and negative association.
